# Approaches to High-Throughput Analysis of Cardiomyocyte Contractility

**DOI:** 10.3389/fphys.2020.00612

**Published:** 2020-07-08

**Authors:** Peter T. Wright, Sharmane F. Tsui, Alice J. Francis, Kenneth T. MacLeod, Steven B. Marston

**Affiliations:** Myocardial Function, National Heart and Lung Institute, Imperial College London, London, United Kingdom

**Keywords:** cardiomyocytes, high-throughput, sarcomere, calcium, contractility

## Abstract

The measurement of the contractile behavior of single cardiomyocytes has made a significant contribution to our understanding of the physiology and pathophysiology of the myocardium. However, the isolation of cardiomyocytes introduces various technical and statistical issues. Traditional video and fluorescence microscopy techniques based around conventional microscopy systems result in low-throughput experimental studies, in which single cells are studied over the course of a pharmacological or physiological intervention. We describe a new approach to these experiments made possible with a new piece of instrumentation, the CytoCypher High-Throughput System (CC-HTS). We can assess the shortening of sarcomeres, cell length, Ca^2+^ handling, and cellular morphology of almost 4 cells per minute. This increase in productivity means that batch-to-batch variation can be identified as a major source of variability. The speed of acquisition means that sufficient numbers of cells in each preparation can be assessed for multiple conditions reducing these batch effects. We demonstrate the different temporal scales over which the CC-HTS can acquire data. We use statistical analysis methods that compensate for the hierarchical effects of clustering within heart preparations and demonstrate a significant false-positive rate, which is potentially present in conventional studies. We demonstrate a more stringent way to perform these tests. The baseline morphological and functional characteristics of rat, mouse, guinea pig, and human cells are explored. Finally, we show data from concentration response experiments revealing the usefulness of the CC-HTS in such studies. We specifically focus on the effects of agents that directly or indirectly affect the activity of the motor proteins involved in the production of cardiomyocyte contraction. A variety of myocardial preparations with differing levels of complexity are in use (e.g., isolated muscle bundles, thin slices, perfused dual innervated isolated heart, and perfused ventricular wedge). All suffer from low throughput but can be regarded as providing independent data points in contrast to the clustering problems associated with isolated cell studies. The greater productivity and sampling power provided by CC-HTS may help to reestablish the utility of isolated cell studies, while preserving the unique insights provided by studying the contribution of the fundamental, cellular unit of myocardial contractility.

## Introduction

The isolation and study of cardiomyocytes have made a remarkable contribution to our understanding of myocardial physiology and pathophysiology. Cardiomyocytes are the elementary units responsible for the contraction of the myocardial tissue. They contain the complete excitation–contraction coupling system and an intact sarcomeric contractile apparatus involved in cellular contraction (Bers, 2002). The isolation of cardiomyocytes makes the study of these processes possible by virtue of extracting the cells from their integrated multi-cellular environment (Harding et al., 1992). The separation of cells from their contractile, vascular, and neurohormonal milieu means that the effects of specific pharmacologic, genetic, or surgical manipulation on cellular physiology can be observed (Davia et al., 1999; Stark et al., 2004; Lyon et al., 2011). From a technical perspective, a single cell is amenable to microscopic analysis. In the case of fluorescent or super-resolution approaches, isolation of cells allows the visualization of cells and sub-cellular/organellar compartments without unwanted effects from the tissue level (Nikolaev et al., 2010; Brandenburg et al., 2016). In recent decades, the genetic manipulation of cells in culture, the introduction of dyes (for Ca^2+^ in particular), and Förster resonance energy transfer (FRET)-based biosensors have become conventional in the isolated cellular preparation (Glukhov et al., 2015; Miragoli et al., 2016; Sanchez-Alonso et al., 2016; Wright et al., 2018a, b).

The very strengths of the isolated cardiomyocyte preparation are unfortunately also a source of weakness. The enzymatic digestion of the heart is not always consistent and batch effects will be observable even if the protocol is run by an experienced operator. From a scientific perspective, this injects variability into datasets, which makes physiologically important effects harder to detect. The separation of cells from their integrated conformation within tissue denies them the support system to which they are evolutionarily adapted. Prolonging the lifetime of acutely dissociated cardiomyocytes by cell culture is artificial and produces time-sensitive preparations in which cells rapidly remodel and de-differentiate even under optimal conditions (Pavlović et al., 2010; Abi-Gerges et al., 2013). The mechano-electric environment is intrinsic to the control of single-cell behavior, and this is technically difficult to replicate for a single-cell preparation. From a statistical perspective, these preparations are “hierarchically clustered” whereby cells from one heart isolation are innately more similar than those isolated from another. Inappropriate analyses that ignore the dependence or “nesting” of measurements of cells from a single preparation are conventionally employed and can lead to “pseudoreplication,” increasing the chance of false positives (Sikkel et al., 2017).

Latent cardiomyocyte heterogeneity (in healthy and failing organs) is an important limitation of single-cell approaches (Paur et al., 2012; Molina et al., 2014; Yue et al., 2017; Wright et al., 2018a). Moreover, the innate electrophysiological differences between myocardial chambers and layers are detailed in the most basic cardiovascular textbook (Szentadrassy et al., 2005) but are usually overlooked as an inconvenient truth by most of those who perform isolated cardiomyocyte studies. In the case of the single-cell preparation, the sampling power of most myocardial techniques are simply too low to make full description of the diversity of cardiomyocyte phenotypes feasible. A view of cardiomyocytes as a monolithic population of cells of a single phenotype persists.

It is evident that the key problem is that current techniques for studying cardiomyocytes are extremely time-consuming while viable cells have a lifetime of hours. Consequently, only a few cells can be studied from each preparation and most remaining cells are wasted. With a small number of cells assayed and the necessity (from ethical, financial, and personnel considerations) to combine measurements from multiple myocyte preparations, this technique may justifiably be challenged as lacking the necessary statistical power to test hypotheses. If it was possible to speed up measurements by assessing in parallel many cells it may be possible to rehabilitate single-cell studies.

Increased throughput measurements can be made possible by reorganizing the microscopy system so that a computer-controlled scanning microscope makes measurements on many myocytes in a stationary sample. In this paper, we report our experiences using the CytoCypher/IonOptix MultiCell System^TM^ to measure myocyte contractility and Ca^2+^ transients. Using this system, operators can expect to measure 30 cells in less than 8 min, with optimal cell function and environmental conditions. With the automated cell finding system, still higher measurement rates are possible and selection bias is reduced (Helmes, 2017). We show how this instrument enables far richer datasets to be collected from a single animal in a short time while cells remain viable. These are more representative of the entire population of cells present in the native myocardial sample enabling us to analyze in depth the statistics and power of the myocyte contraction measurement. In addition, we demonstrate high-throughput routines to investigate the pharmacological profiles of known drugs that act upon the contractile apparatus.

## Methods and Equipment

### Animal Care and Procedures

Animal experiments met the criteria of Imperial College London and Animals in Scientific Procedures Act (ASPA) 1986 as well as the 2010/63/EU Directive.

**Instrumentation:** 

**Module**	**Manufacturer**
CytoCypher Microscope Module with 35 mm Dish Insert	CytoCypher BV, Netherlands
CytoCypher System Control Module	CytoCypher BV, Netherlands
IonOptix MyoCam S3 Video Camera	IonOptix LLC, United States
Myopacer with 35 mm Dish Field Stimulation Element	IonOptix LLC, United States
UV LED Fluorescent Source	Cairn Research, United Kingdom
UV Filter Set	Cairn Research, United Kingdom
IonOptix Fluorescence System Interface	IonOptix LLC, United States
Cell Framing Adaptor	IonOptix LLC, United States
Desktop PC	
CytoCypher Perfusion Module	CytoCypher BV, Netherlands
(optional)	

**Software:** 

**Software**	**Developer**
IonOptix IonWizard	IonOptix LLC, United States
CytoCypher TAT	CytoCypher BV, Netherlands
CytoCypher Cytosolver	CytoCypher BV, Netherlands
Cytospectre	University of Tampere, Finland

### Myocyte Isolation Protocols

#### Rat Isolation

Rats are sacrificed by cervical dislocation after the induction of a brief anesthesia in an atmosphere of 5% isoflurane. An incision is made across the base of the rib cage and on either side of the thorax to fully expose the thoracic cavity. The entire contents of the cavity are removed and placed in ice-cold Krebs–Henseleit (KH) Buffer (119 mM NaCl, 4.7 mM KCl, 0.94 mM MgSO_4_, 1 mM CaCl_2_, 1.2 mM KH_2_PO_4_, 25 mM NaHCO_3_, and 11.5 mM glucose; 95% O2, 5% CO_2_). The tissue is placed in clean ice-cold KH. The lungs, thyroid, and superfluous pericardial fat are dissected away from the heart, leaving the aorta and atria intact. The heart is then rapidly cannulated via the aorta, connected to a Langendorff apparatus and retrogradely perfused with KH at 37°C. After 1–1½ min, the KH buffer is switched to a low Ca^2+^ (LoCa^2+^) buffer [12–15 μM CaCl_2_, 120 mM NaCl, 5.4 mM KCl, 5 mM MgSO_4_, 5 mM pyruvate, 20 mM glucose, 20 mM taurine, 10 mM HEPES, and 5 mM nitrilotriacetic acid (NTA); 100% O_2_] for 5 min. Subsequently, a solution of 1 mg/ml Collagenase II and 0.6 mg/ml Hyaluronidase (C + H) in enzyme (Enz) buffer (120 mM NaCl, 5.4 mM KCl, 5 mM MgSO_4_, 5 mM pyruvate, 20 mM glucose, 20 mM taurine, 10 mM HEPES, and 150 μM CaCl_2_) is perfused into the heart for 10 min. After 10 min of enzymic digestion, the Langendorff perfusion is switched off and the heart is cut down. The heart is then placed in fresh C + H and dissected and minced. The samples are gently shaken mechanically at 35°C for 5 min and filtered through gauze. If necessary, the right and left ventricles are separated and processed separately. The undigested tissue is placed back in the tube with fresh C + H and shaken for a further 30 min. The solution is then strained through gauze and the resulting filtrate is centrifuged for 1 min at 700 rpm to yield a pellet of cells. These isolations yield between 80 and 95% rod-shaped cardiomyocytes.

#### Mouse Isolation Procedure 1

Mouse cells are isolated using one of two different procedures. The first is similar to that described for rat with several modifications. The mouse heart is extracted using the same procedure as for rat and placed in cold KH buffer. The heart is dissected and retrogradely perfused on a modified Langendorff apparatus with KH for 1–1½ min. Subsequently, the heart is perfused with the LoCa^2+^ buffer for 5 min. Following this stage, protease solution (0.36 mg/ml) is perfused for 1 min before switching to C + H for 5 min. The heart is then dissected in Enz and passed through a pipette to break up the tissue and yield single cells.

#### Mouse Isolation Procedure 2

In the second procedure, the mouse heart is initially placed into ice-cold HEPES buffer (NaCl 113 mM, KCl 4.7 mM, KH_2_PO_4_ 0.6 mM, Na_2_HPO_4_ 0.6 mM, MgSO_4_ 1.2 mM, NaHCO_3_ 12 mM, KHCO_3_ 10 mM, HEPES 10 mM, and Taurine 30 mM). It is then perfused with the HEPES buffer at 37°C, after the fluid exiting the heart becomes clear, trypsin/Liberase solution is added to the perfusate (Solution composition as detailed above plus 13 μM CaCl_2_, Liberase 0.05 mg/ml, and 0.3 mg/ml Trypsin). The heart is then dissected in perfusion buffer and passed through a pipette to break up the tissue and yield single cells.

These isolations yield between 60 and 80% rod-shaped cardiomyocytes.

#### Guinea Pig Isolation Procedure

The guinea pig heart is placed in ice-cold heparinized KH buffer (NaCl 120 mM, KCl 4.7 mM, MgSO_4_ 0.94 mM, KH_2_PO_4_ 1.2 mM, NaHCO_3_ 25 mM, Glucose 11.5 mM, and CaCl_2_ 1 mM, pH 7.4). The heart is perfused with KH 1–2 min at a rate of 6–8 ml/min. A low-Ca^2+^ (LoCa^2+^ NaCl 120 mM, KCl 5.4 mM, MgSO_4_ 5 mM, Na Pyruvate 5 mM, Glucose 20 mM, Taurine 20 mM, HEPES 10 mM, CaCl_2_ 12–15 μM, NTA 5 mM, and pH 6.96) is perfused for 4–5 min; all cardiac contraction ceases. An enzyme solution containing Liberase TL is perfused for 6–10 min [NaCl 120 mM, KCl 5.4 mM, MgSO_4_ 5 mM, Na Pyruvate 5 mM, Glucose 20 mM, Taurine 20 mM, HEPEs 10 mM, CaCl_2_ 0.2 mM, and Liberase TL (Gibco) 0.1 mg/ml]. The left ventricle is then dissected into 2-mm^3^ cubes. The chunks are added to the same enzyme solution, shaken, oxygenated, and triturated. The solution is filtered every 3 min until digestion complete. The suspension is centrifuged at 400 rpm for 1 min and the resulting cell pellet is resuspended in fresh enzyme solution without Liberase TL. Myocytes are stored in DMEM solution at room temperature and ready for experimental use.

These isolations yield between 70 and 85% rod-shaped cardiomyocytes.

#### Human Cardiomyocyte Isolation Procedure

Samples of human atria or ventricles are obtained from consenting patients within the Imperial College Healthcare Trust heart transplant program in accordance with the Declaration of Helsinki and local ethical review. Biopsies of human tissue are acquired and placed in ice-cold Low Ca^2+^ solution [NaCl 120 mM, KCl 5.4 mM, MgSO_4_ 5 mM, Pyruvate 5 mM, Glucose 20 mM, Taurine 20 mM, HEPES 10 mM, and NTA 5 mM, bubbled with 100% O_2_. pH was adjusted to 6.95, free [Ca^2+^] was 1 to 3 μM]. The tissue is diced into 1-mm^3^ chunks in ice-cold LoCa^2+^. It is then washed for 3 min in warmed, oxygenated LoCa^2+^ by manually shaking in a water bath; after the first step, the supernatant is removed and replaced with fresh LoCa^2+^ and shaken again.

##### Ventricular tissue

Subsequently, the tissue is shaken for 45 min in enzyme solution containing protease XXIV (NaCl 120 mM, KCl 5.4 mM, MgSO_4_ 5 mM, pyruvate 5 mM, glucose 20 mM, taurine 20 mM, HEPES 10 mM, 150 μM CaCl_2_, and protease 4 U/ml–0.36 mg/ml). The supernatant is removed by filtering through gauze, and the undigested tissue is then shaken for 10–15 min in solution containing collagenase Type V (NaCl 120 mM, KCl 5.4 mM, MgSO_4_ 5 mM, pyruvate 5 mM, glucose 20 mM, taurine 20 mM, HEPES 10 mM, 150 μM CaCl_2_, and collagenase 1 mg/ml). This step is repeated multiple times. After each stage, the supernatant is filtered through gauze and the undigested tissue is returned to the shaker bath. Supernatants are centrifuged at 700 rpm for 2 min. Cell pellets are resuspended in enzyme solution without enzymes.

##### Atrial tissue

This procedure is analogous to the isolation of ventricular tissue except that the protease containing solution is supplemented with type V collagenase (collagenase, 0.5 mg/ml).

These isolations yield between 10 and 25% rod-shaped cardiomyocytes.

### Cell Culture

#### Laminin Attachment

Isolated cells are plated on laminin-coated coverslips or Matek^TM^ (Matek Corp, United States) dishes, in culture media containing FBS (MEM salts 10.74 g/L, NaHCO_3_ 9 mM, L-Glutamine 1%, and Penicillin/Streptomycin 1%). Cells are incubated for 1 h to allow attachment (5% CO_2_, 37°C). The dishes are then supplemented with 1 ml of MEM to prevent drying.

#### Free Solution

Isolated cells are resuspended in MEM solution and plated in 35-mm petri dishes with 13-mm glass inserts and placed in the incubator (5% CO_2_, 37°C) until use.

#### Fura-2 or Fura-4f AM Loading

Fura-2AM or Fura-4fAM are resuspended in DMSO to produce 1 mM solutions. Cells attached to laminin are loaded by applying perfusion solutions containing 1 μM or 5 μM Ca^2+^-sensitive dye to Matek and incubated in the dark at room temperature for 10–15 min or 25 min for guinea pig cells. Cells in solution are aspirated, spun gently, and resuspended in perfusion solutions containing 1 μM Fura-2/Fura-4fAM. The solutions are spun again and the dye containing solution is removed. The cells plated on Matek or in solution are washed once with clean perfusion buffer before analysis on the CytoCypher system.

### Measurements With CytoCypher HTS

**FIGURE 1:**
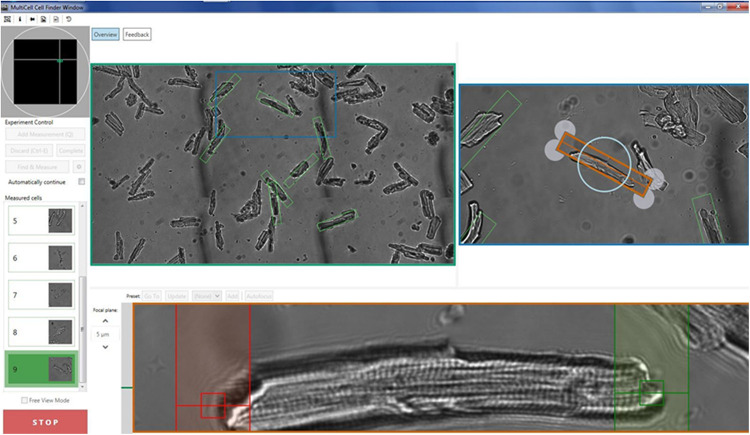
Illustration of the acquisition screen of the CytoCypher High-Throughput System for the analysis of cardiomyocyte contractility. See also Supplementary Video.

Isolated cardiomyocytes are paced using a field stimulator at a frequency of between 0.2 and 2 Hz (20–50 V, 10-ms pulse width). The CytoCypher HTS is based on a static observation platform for the cells and a static high-resolution camera with a scanning microscope taking advantage of the infinity corrected optics present in most modern microscope lenses. This allows for rapid scanning without disturbing the cells. The area that can be scanned is 15 mm^2^. A mosaic field approximately 3500 × 1600 μm is presented from which a region of interest can be selected (Figure 1), and individual cells were located and orientated. The selected cell is optimized for sarcomere length detection and then the recording can be made. The cell is marked for re-assay by the software with the coordinates of where the measurement was taken marked out with a green rectangle. The next cell is subsequently selected. Microscope movements are virtually instantaneous, and position can be refined by mouse or keyboard control, taking just a few seconds. Experienced operators can expect to measure up to 30 cells in <8 min. An automated cell finding system enables automatic detection and measurement of myocytes (Supplementary Video 1). In favorable conditions, this is quicker than the best manual selection. Since selection is based on pre-defined criteria, results obtained by the system itself cannot be influenced by operator bias, for instance, by selecting the most obviously contractile cells (Table 1). For Fura-dye measurements, the microscope is supplemented by a dual excitation LED light source and photomultiplier detector for simultaneous ratiometric Ca^2+^ measurements (this solid-state solution is a considerable advance on current rotating filter methodology). Data are analyzed using the Cytosolver or Transient Analysis Tool (IonOptix LLC, United States) software environments; these packages are a development of the well-established IonWizard software with much greater speed and a user-friendly interface. For the purposes of this study, we have analyzed three key parameters of contraction magnitude and speed: percentage shortening of sarcomeres, time to peak from 10% of peak (TTP90), and time from peak to 10% above baseline (TTB90).

**TABLE 1:** Standard cellular morphological parameters utilized for manual and automated cell finding by the CytoCypher system.

**Manual acquisition criteria**	
1. Rod shaped morphology with clear and well-organized sarcomere striations throughout the cell body. Cell bodies with artifacts or abnormal bulges were avoided.	
2. Regular contractions upon electrical stimulation. Exclude myocytes with extra or escaped beats	

**Automatic acquisition criteria**	
**Area**	
Maximum area (μm^2^)	6000
Minimum area (μm^2^)	1000
**Aspect ratio**	
Maximum ratio	0.5
Minimum ratio	0.1
**Misc.**	
Cell Overlap Parameter (μm; i.e., avoids two cells together)	40
Number of cells	50
Well scanning	Spiral motion
Operations on full field	Static cell finding with autofocus

### Morphological Analysis of Cardiomyocytes

**FIGURE 2:**
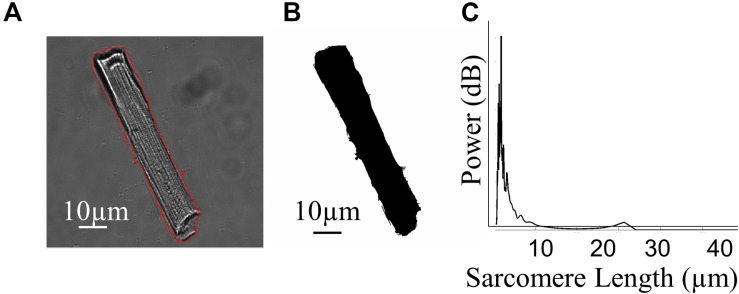
Morphological investigation of cardiomyocytes. **(A)** Example image of a rat cardiomyocyte acquired by the CytoCypher HTS during the measurement of contraction; the red line indicates the application of a segmentation mask. **(B)** Segmentation mask generated automatically by Fiji software using our semi-automated analysis. **(C)** Plot demonstrating the relative “power” of different frequencies within the cell, generated by the Cytospectre program.

As well as functional data, the CytoCypher HTS acquires thumbnail images of each cell it investigates (Figure 2A). This allows one to derive 2D morphological information. We create a segmentation mask using a self-produced macro in FIJI (Figure 2B) and use this in conjunction with the freeware image analysis program Cytospectre (University of Tampere, Finland; Kartasalo et al., 2015). As well as measuring normal geometric parameters from the segmentation mask, this program performs a spectral analysis of the region of interest and can give an indication of the dominant frequencies within the entire cardiomyocyte. Figure 2C shows the mixed coarse frequency component of the image of a cardiomyocyte; the repeating sarcomere structures within the cell dominate and give a large peak at around 1.8 μm. A more organized cell with a stronger sarcomere pattern will have a taller, sharper peak at the mode frequency relating to the average sarcomere length. Therefore, one can analyze the nature of the distribution of these geometric and spectral parameters alongside functional ones within a given cell sample and adapt statistical methodology accordingly.

### Statistics

As discussed in the Introduction section, we believe that conventional analyses that utilize single-cell recordings as independent samples commit the error of “pseudoreplication,” introducing a significant false-positive rate in studies of isolated cells (Sikkel et al., 2017). Conventional *t* tests and analysis of variance assume that each sample is independent and combine data from many preps, but in reality, cells from one preparation are more like each other than cells from other preparations. As a result, individual cell recordings taken from the same preparation can be nested within that preparation, meaning that a hierarchical structure is present within the dataset. In this paper, we use GraphPad Prism 6.0 to compute conventional column statistics from datasets without introducing hierarchical structure (mean, standard deviation). When comparing two independent conditions, we used unpaired Students’ *t* test assuming statistical significance if *p* < 0.05. Where pharmacological stimuli were applied to a preparation of cells after baseline measurements were taken, we used a paired *t* test assuming statistical significance of *p* < 0.05. If data were not normally distributed, we applied Wilcoxon’s test. To perform an analysis of the datasets with hierarchical structure preserved, we used a pre-published methodology using RStudio (Sikkel et al., 2017). Sikkel et al. provide a full explanation of the theoretical issues of this area and the practical implementation of hierarchical techniques. Their method produces a theoretical mean and standard error based on the relative contribution of individual preparations to the totality of the data. It also gives an intra-class correlation (ICC) statistic that indicates the similarity of preparations also known as “clustering.” If the mean value of samples is very different between preparations with little overlap in the distributions of values, then the data are considered highly clustered with a high ICC. Complete overlap of the distributions of all preparations and identical means will yield an ICC of 0%. Based on these statistics, this analysis produces a *t* test (plus a relevant *p* value) between condition A and B using the theoretical means, errors, and idealized degrees of freedom and indicates whether a hierarchical or conventional statistical approach is most suitable. All statistical tests are indicated in Figure legends * *p* < 0.05, ** *p* < 0.001, *** *p* < 0.001, and **** *p* < 0.0001.

## Results

### A New Paradigm for Measuring Myocyte Contractility

The principles for high-throughput myocyte measurements differ radically from previous methods. We study many cells, each for a short period rather than a few cells over extended periods. Myocytes are plated onto laminin-coated 35-mm dishes and mounted in the CytoCypher apparatus (Figure 1 and Supplementary Video 1). Measurements are made on as many of the myocytes in a field of view as necessary. In practice, this can be between 10 and 50 myocytes. Measurements are made by sarcomere length detection using the Fast Fourier Transform method or by cell length based on edge detection if the sarcomere pattern is not sufficiently clear. Myocytes may be selected manually using mouse and keyboard controls to locate, focus, and orientate the myocyte in about 4 s. A short recording of contractions, usually over a period of ∼5–10 s (depending on stimulation frequency) is made before moving on to the next cell.

In automatic mode, the microscope searches for objects that meet the preset criteria (Table 1). This mode is preferable since it avoids operator bias. It is optimal with about 20 viable myocytes in a field of view; sparse fields of myocytes can be detected more quickly manually. The data are then saved and may be analyzed online using Cytosolver software or offline using the Transient Analysis Tool, yielding the same contractility parameters as the well-known IonWizard software but much more rapidly. This avoids moving the bottleneck from the acquisition to the analysis phase. The myocyte positions are saved so that the myocytes may be remeasured; in this way, it is possible to build up a time course of parameters for individual cells (see Figure 1).

Although changing solution in a flow cell is possible, this would slow down the measurements in the CytoCypher. Our usual protocol is to aspirate the fluid from the dish of myocytes and replace it with a new solution, an operation that takes just a few seconds. It is possible to make up to four solution changes this way before the cell population becomes too sparse. Alternatively, one could use a fresh dish of myocytes for each solution change.

**FIGURE 3:**
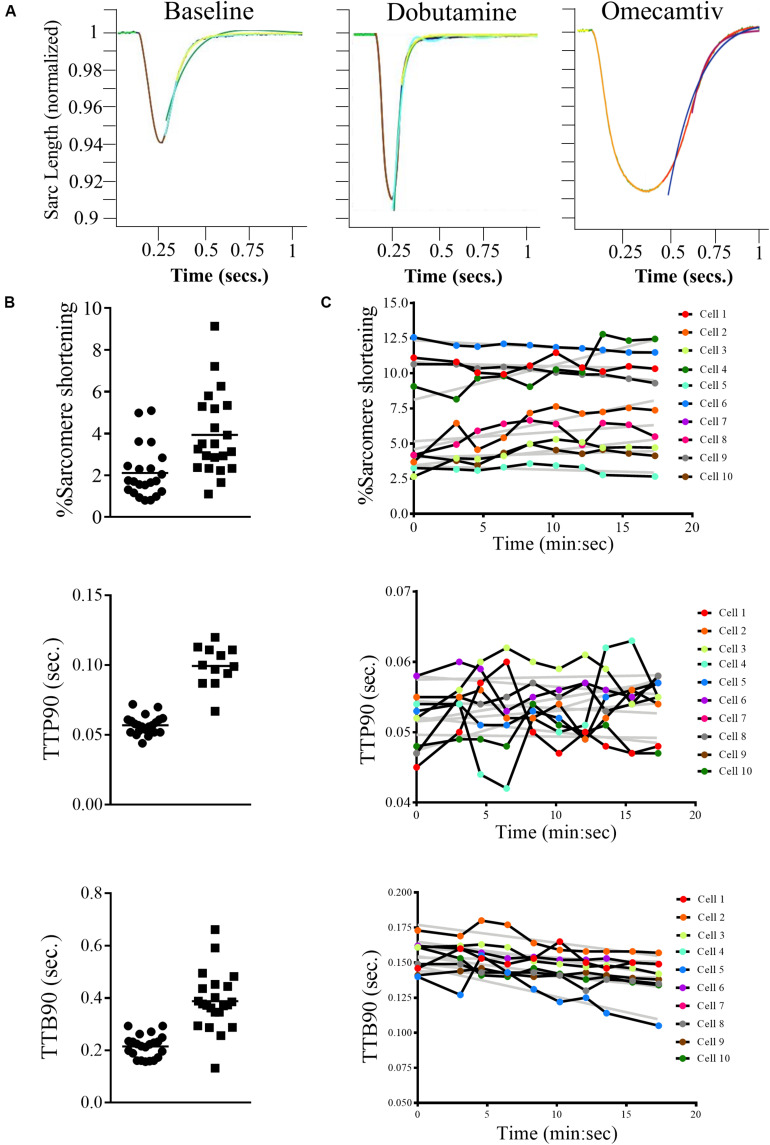
Examples of measurements made using the CytoCypher HTS. **(A)** Average rat sarcomere length transients, 1 Hz, 20 V stimulation; baseline, 3 μM dobutamine (note larger peak and faster transients), and 2.4 μM Omecamtiv Mecarbil (note slower transients). The traces are averages of 10 contractions collected over 10 s. The colored segments represent green original average trace; yellow, baseline fit; orange, peak fit; red, recovery fit; purple, single exponential fit (Tau); and blue, double exponential fit. **(B)** The calculated parameters,% shortening, TTP90, secs and TTB90, secs from approximately 20 myocytes at baseline and in the presence of 2.4 μM Omecamtiv Mecarbil are shown with mean value indicated. Automatic cell finding; the two measurements took about 4 min each. Distribution of values is not normal, so significance is tested by Wilcoxon method. **(C)** Time course of the calculated parameters (% shortening, TTP90, secs and TTB90, secs) in the presence of 3 μM dobutamine + 0.05 μM ICI 118,551 for 10 myocytes in a field of view over 20 min. The gray lines are linear regression fits to the data. This plot shows the variability, both between myocytes and its change over time. The values in individual myocytes may increase or decrease slightly with time but on average% shortening and TTP90 are stable while TTB90 declines slightly over time.

For example, we measured the sarcomere contraction response of three dishes of cells, one untreated, one treated with the β-adrenoceptor agonist dobutamine, and one treated with the myosin modulator Omecamtiv Mecarbil (OM; Figure 3A). The dobutamine increases the amplitude of contraction and shortens the relaxation, whereas OM has the opposing effect. Figure 3B presents scatter plots of the recordings of percentage sarcomere shortening, time taken to 90% peak contraction from baseline (TTP90), and time taken to 90% relaxation (TTB90) following treatment with OM (2.4 μM). Figure 3C presents time course data for the three parameters following individual cells over the course of their treatment with resampling at 2.5-min intervals.

### Does Fura-4f Treatment Affect Myocyte Contractility?

#### Analysis of Hierarchical Clustering in Parameters Relating to Cardiomyocyte Function

Researchers may desire data concerning Ca^2+^ handling from cells that have been labeled with a Ca^2+^ dye such as Fura-2. Unfortunately, high-affinity Ca^2+^dyes have been reported to buffer Ca^2+^ within the cell and alter their physiological behavior (Wokosin et al., 2004; Fast, 2005). Here, we demonstrate baseline contraction data for cells with and without loading with Ca^2+^ dye Fura-4f. In populations of cells that are not loaded with Fura-4f, about 20–30% appear to be non-contractile (Supplementary Figure 2A). By contrast, in sets of cells labeled with Fura-4f, 10–15% are not contractile (Supplementary Figure 2B).

**FIGURE 4:**
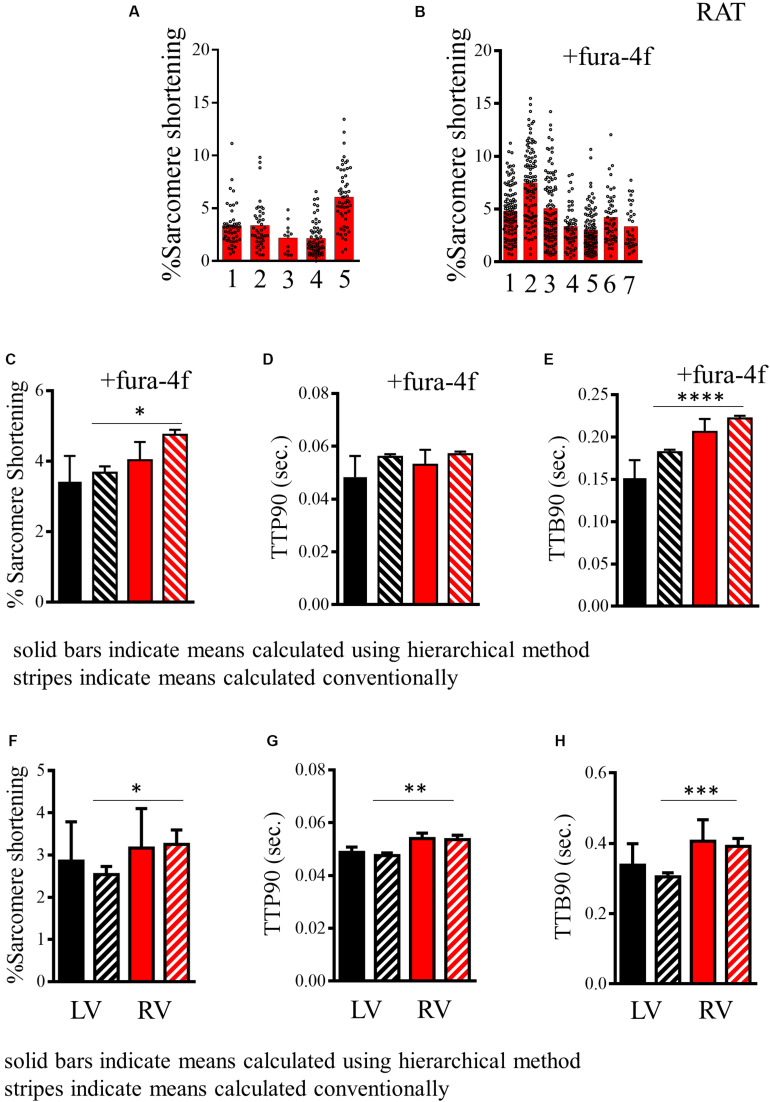
Analysis of contractility of rat cardiomyocytes. **(A,B)** Histogram displaying measurements of the baseline sarcomere shortening of multiple individual preparations of rat cardiomyocytes without and with fura-4f loading. **(C)** Histogram comparing estimated mean and standard deviation of sarcomere shortening in the populations of rat cardiomyocytes presented in A (–Fura-4f black) and B (+Fura-4f red) with hierarchical structure included (solid bars) and assuming all measurements are independent samples (striped bars). **(D)** Histogram comparing estimated mean and standard deviation of TTP90 in populations of rat cardiomyocytes presented in Supplementary Figure 2C (–Fura-4f black) and D (+Fura-4f red) with hierarchical structure included (solid bars) and assuming all measurements are independent samples (striped bars). **(E)** Histogram comparing estimated mean and standard deviation of TTP90 in populations of rat cardiomyocytes presented in Supplementary Figure 2E (–Fura-4f black) and F (+Fura-4f red) with hierarchical structure included (solid bars) and assuming all measurements are independent samples (striped bars). **(F)** A representation of the estimated mean and standard error of sarcomere shortening of left and right ventricular cardiomyocytes isolated from mice. Datasets assuming a hierarchical structure (solid bars) and assuming no structure (striped bars). **(G)** Representations of the means and standard error of TTP90 of left and right ventricular cardiomyocytes isolated from mice with hierarchical structure (solid) and without (striped). **(H)** Representation of the means and standard error of TTB90 of left and right ventricular cardiomyocytes isolated from mice with hierarchical structure (solid) and without (striped).

As stated earlier, many indices of cardiomyocyte function are not truly independent measurements but are “nested” within a population derived from a specific animal. Cells from the same isolation are more alike and “cluster” around a specific mean for that preparation. In Figure 4, we present the baseline data for a group of five to seven independent rat cardiomyocyte isolations derived from measures of sarcomere shortening recorded in the presence of 2 mM Ca^2+^ and stimulated at 1 Hz. As can be seen from the histograms in Figures 4A,B plotting results from different preparations, average sarcomere shortening for each preparation shows significant variation in addition to the variable distribution of the individual myocyte measurements around the preparation mean. We present the mean and standard error for these data computed by conventional methods with each measurement assumed to be independent or with correction for hierarchical effects, for rat cells with or without Fura-4f labeling (Figure 4C). Table 2 displays numeric data with relevant statistics allowing comparison of the two datasets. Our analysis indicates an ICC score of 18.5% for % sarcomere shortening for our populations of cells. This suggests a moderate level of clustering, which is observable in the histograms. The common unpaired *t*-test indicates that the difference between the two groups is statistically significant (*p* < 0.016). In Figure 4F, we indicate the hierarchical estimate mean and standard error of the two cell populations. Such statistical treatment reveals that there is no difference in the means of the two groups (*p* = 0.51; Table 2). The effective degrees of freedom due to hierarchical effects computed by our analysis are 12.1 and 12.3 for the respective samples, indicating that the statistical significance suggested by the common test is the result of significant “pseudo-replication.” This analysis indicates that statistical methods acknowledging “hierarchy” are required to firmly test any hypothesized difference between Fura-4f loaded cells and control.

**TABLE 2:** Table describing the mean and standard error of baseline measurements of myocytes from mouse, rat, guinea pig, and human hearts measurements of functional parameters.

			**Simple statistics**			**Hierarchical statistics**				***p* values**

**Sample**	**Prep.**	***n***	**%**	**TTP90**	**TTB90**	**% (ICC)**	**TTP90 (ICC)**	**TTB90 (ICC)**	**Suit.**	***p* values**
Rat	5	226	3.670 ± 0.180	0.056 ± 0.001	0.182 ± 0.003	3.148 ± 0.730 (18.5)	0.048 ± 0.008 (28.2)	0.151 ± 0.021 (16.7)	YYY	Rat vs. rat + fura
Rat + Fura	7	510	4.751 ± 0.137	0.057 ± 0.001	0.222 ± 0.003	4.026 ± 0.518	0.053 ± 0.006	0.206 ± 0.015		parameter Simple Hierarchical
										% < 0.0001 0.051
										TTP90 0.55 0.64
										TTB90 < 0.0001 0.06
Mouse LV	3	165	2.539 ± 0.191	0.048 ± 0.001	0.305 ± 0.012	2.854 ± 0.930 (30.9)	0.049 ± 0.002 (3.4)	0.338 ± 0.006 (30.3)	YNY	Mouse LV vs. mouse RV
Mouse RV	3	84	3.250 ± 0.346	0.054 ± 0.002	0.391 ± 0.023	3.164 ± 0.937	0.054 ± 0.002	0.406 ± 0.061		parameter Simple Hierarchical
										% 0.0519 0.82
										TTP90 0.0013 0.15
										TTB90 0.0002 0.46
Guinea Pig	1	71	5.213 ± 0.360	0.228 ± 0.010	0.627 ± 0.021	–	–	–	–	–
Human LV	1	4	3.202 ± 0.994	0.244 ± 0.040	0.687 ± 0.074	–	–	–	–	–
Human LA	1	14	0.430 ± 0.161	0.062 ± 0.016	0.446 ± 0.153	–	–	–	–	–
Human RA	1	5	2.500 ± 0.832	0.218 ± 0.121	0.637 ± 0.131	–	–	–	–	–

*The table describes the statistics generated by conventional and advanced (hierarchical) approaches and includes the results of statistical significance between appropriate samples. The column (suit.) describes whether the advanced test (*Y*) or the conventional test (*N*) is sufficient in each case. Results from several preparations are presented using simple statistics (mean ± standard deviation) and using hierarchical statistics (mean ± standard deviation and ICC). The *p* values column presents the calculations of *p* using Student’s *t* test or the hierarchical method. Significance is commonly reduced using hierarchical statistics.*

Similar clustering can be observed for the time taken to reach 90% of the peak from baseline (ICC, 28.2% TTP90; Supplementary Figures 2C,D). There is no difference between the means estimated for cells with and without Fura-4f loading (Figure 4D and Table 2). For this parameter, both the common test and the hierarchical test indicate no significant difference (*p* = 0.11 and 0.64, respectively).

Loading cells with Fura-4f appears to lengthen the time taken to reach 90% of the baseline (TTB90), indicating slower relaxation. Clustering can be seen within the different batches of cells (ICC, 16.7%; Supplementary Figures 2E,F). The common test suggests that the increase in TTB90 is statistically significant (*p* < 0.0001). The hierarchical estimate means also indicate a longer TTB90 for Fura-4f-loaded cells, but this difference just falls short of statistical significance (*p* = 0.06, df 11.1–10.9; Figure 4E and Table 2). Once again, the difference indicated by the common test is the result of a large amount of pseudo-replication, with each measurement from a single preparation only accounting for around two theoretical degrees of freedom.

### Comparisons of Mouse, Guinea Pig, and Human Myocytes With Rat

#### Mouse Cardiomyocytes

The mouse is a common model species employed in cardiovascular biology, but the isolation of cardiomyocytes from this animal is more technically challenging than from the rat. Here, we present baseline data derived from measurements of changes in sarcomere length of cells from the left and right ventricles of three individual mice. Greater clustering can be observed for each of the parameters reflecting greater batch-to-batch variability in the cells. This appears to account for most of the variation because cells from left and right ventricles from the same heart seem to be more similar than cells from the same region of different hearts (Supplementary Figures 3A–C). Measurements of % sarcomere shortening are moderately clustered (ICC, 30.9%) for left and right ventricular cells, meaning hierarchical statistical methods must be employed (Table 2). The analysis we perform applies a test to determine whether hierarchical or common statistical approaches should be employed. Cardiomyocytes derived from right ventricle seem to have a slightly greater mean baseline contractility. The common unpaired *t*-test suggests that this is statistically significant (*p* < 0.05). However, this is not the case for the estimated means due to the correction for pseudoreplication (*p* = 0.822, df < 6; Figure 4F and Table 2).

Measurements of TTP90 display very little clustering (ICC, 3.4%), the hierarchical analysis is not strictly necessary here. Population and estimated means suggest a longer TTP90 in right ventricular cells. An unpaired *t*-test indicates that this is statistically significant (*p* < 0.01), but this disappears after hierarchical correction (*p* = 0.15, df < 3–5; Supplementary Figure 3B).

TTB90 measurements are moderately clustered (ICC, 30.3%; Table 2 and Supplementary Figure 3C). Population and estimated means seem to suggest a slightly longer TTB90 in right ventricular cells on average. The common *t* test indicates that this difference is statistically significant (*p* < 0.001) but again this disappears with correction of clustering and psuedoreplication (*p* < 0.4599, df < 5; Figure 4H). We also present a small amount of data for cells isolated from entire mouse hearts, without dissection and loaded with the Ca^2+^ dye Fura-4f in two isolations. Apparently, Fura-4f slightly reduces contractility and lengthens TTB90. This is of course slightly different from the rat data that utilized Fura-4f (Supplementary Figures 3D–F).

#### Human Cardiomyocytes

**FIGURE 5:**
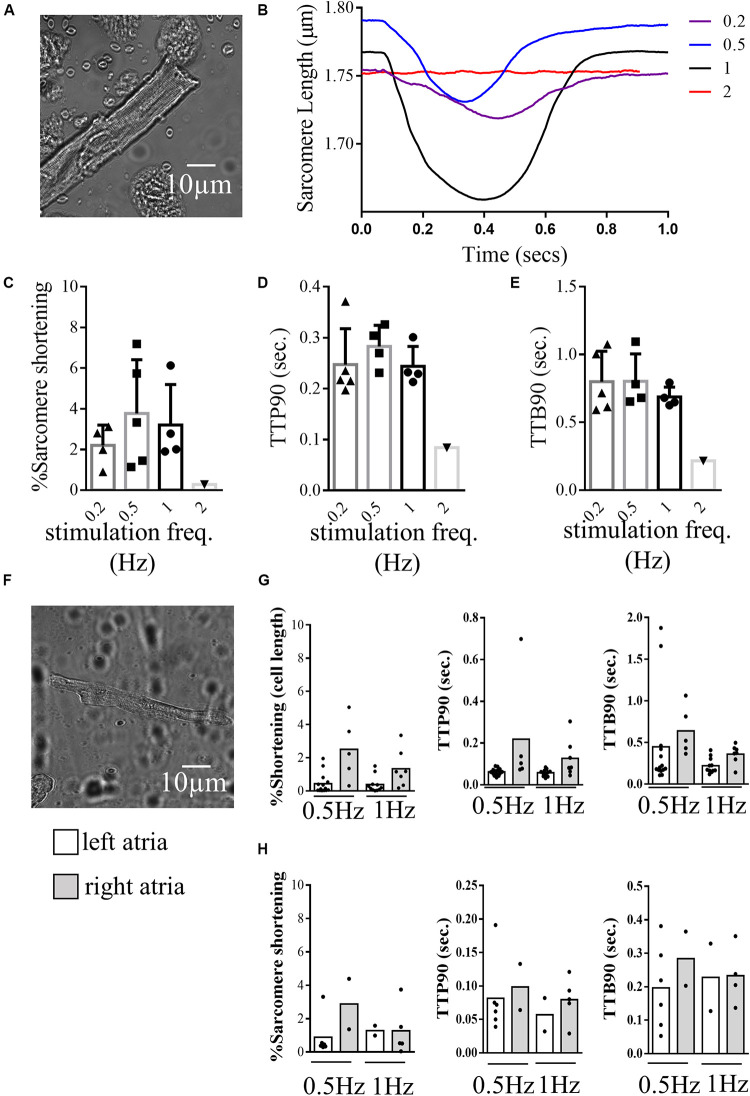
Contractility of human cardiomyocytes. **(A)** Example image of a human ventricular cardiomyocyte isolated from the left ventricle of a patient with hypertrophic cardiomyopathy recorded during contraction analysis by the CytoCypher HTS. **(B)** Example traces of sarcomere shortening during the contraction cycle in the same cell paced at different frequencies. **(C)** Histogram and dot plots demonstrating the percentage sarcomere shortening of ventricular cells at different pacing frequencies. triangle = 0.2 Hz, square = 0.5 Hz, circle (line in bold) = 1 Hz inverted triangle = 2 Hz. **(D)** Histogram and dot plots demonstrating the TTP90 of sarcomere shortening of ventricular cells at different pacing frequencies. **(E)** Histogram and dot plots demonstrating the TTB90 of sarcomere shortening of ventricular cells at different pacing frequencies. **(F)** Example image of a human cardiomyocyte isolated from the left atrial chamber of a patient with hypertrophic cardiomyopathy. **(G)** Histogram and dot plots representing the cell shortening behaviors of cardiomyocytes isolated from the left and right atrial chambers of the same patient, paced at 0.5, and 1 Hz (% cell shortening, TTP90, and TTB90). clear bar = left atria and gray bar = right atria **(H)** Histogram and dot plots representing the sarcomere shortening behaviors of left and right atrial cardiomyocytes isolated from the left and right atria paced at 0.5 and 1 Hz (% sarcomere shortening, TTP90, and TTB90). clear bar = left atria and gray bar = right atria.

The CytoCypher HTS system was able to collect significant amounts of data from myocytes prepared from small human heart biopsies due to its speed and ability to analyze all the cells in a dish. Figure 5 presents measurements from cardiomyocytes isolated from human ventricles and atria explanted from patients with hypertrophic cardiomyopathy. Figures 5A–E show results with a ventricular sample: Figure 5A shows the morphology of the ventricular myocyte in a bright-field thumbnail image acquired by the CytoCypher system. Figure 5B presents plots of sarcomere length over the contraction cycle at different pacing frequencies. For this cell, the largest contraction was observed at 1 Hz. Variation in the baseline contraction can be observed at different pacing frequencies, indicating the sensitivity of this parameter to the experimental conditions. At 2 Hz, the cell became arrhythmic and a transient could not be recorded. Histograms present the average % sarcomere shortening, TTP90, and TTB90 over a range of stimulation frequencies in Figures 5C–E.

Figures 5F–H show results obtained with atrial samples. Figure 5F shows the morphology of a cardiomyocyte from the left atria of a patient with hypertrophic cardiomyopathy. The sarcomeric structure of these cells is less well defined than those from rodent or human ventricle. As a result, cell length measurements were more readily visualized by the CytoCypher than measurements from the sarcomere pattern. Figure 5G shows results using cell length detection and Figure 5H shows the same cells with sarcomere length detection for left and right atria at two stimulation frequencies.

#### Guinea Pig Cardiomyocytes

The guinea pig is a common model animal in fundamental cardiovascular biology. It is particularly useful for electrophysiological studies due to a closer relationship between the guinea pig and human action potential morphology (ion channel complement) than the rat or mouse. In Supplementary Figure 4, we present baseline data for contractility and Ca^2+^ handling in control Dunkin-Hartley guinea pig cardiomyocytes paced at 0.5 Hz. Supplementary Figure 5A presents example traces of guinea pig sarcomere shortening and Ca^2+^ transients in Fura-4f-loaded cells. Percentage shortening, TTP90, and TTB 90 were measured by both sarcomere length and cell length procedures (B–G). These measurements reveal the intimate connection between cell shortening and sarcomere shortening. Although the percentage shortening is similar to the rat and mouse, the guinea pig shows slower contraction kinetics, especially contraction onset as indicated by TTP90 (Table 2).

#### Hierarchical Statistics of Ca^2+^ Transients

**FIGURE 6:**
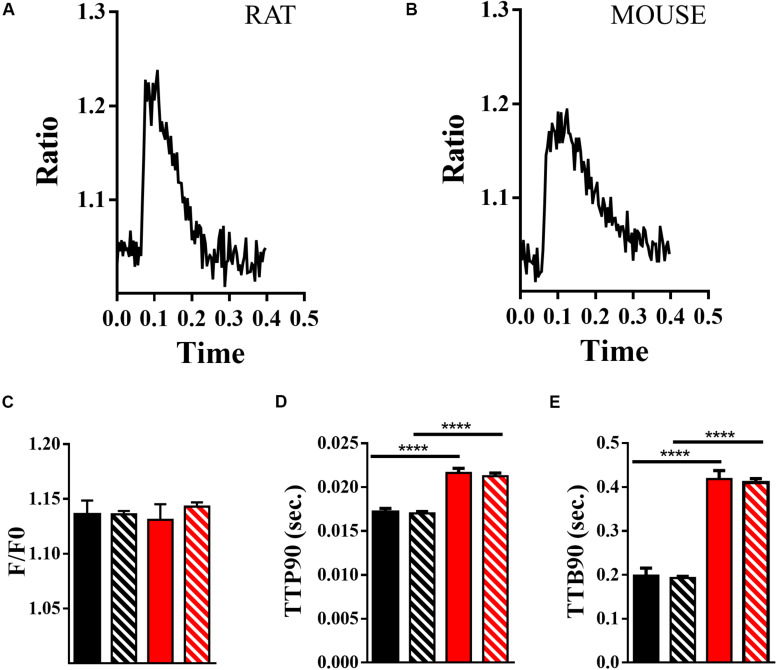
Measurement of Ca^2+^ transients using Fura-4f. **(A)** Example Ca^2+^ transient from a rat ventricular cardiomyocyte loaded with fura-4f. **(B)** Example Ca^2+^ transient of a mouse ventricular cardiomyocyte loaded with fura-4f. **(C)** Comparison of F/F0 of rat (black) and mouse (red) Ca^2+^ transients, means and standard error with hierarchical effects included (solid) and without (striped). **(D)** Comparison of TTP90 of Ca^2+^ transients of rat (black) and mouse (red) Ca^2+^ transients, means and standard error with hierarchical effects included (solid) and without (striped; ****p < 0.0001). **(E)** Comparison of TTB90 of Ca^2+^ transients of rat (black) and mouse (red) Ca^2+^ transients, means and standard error with hierarchical effects included (solid) and without (striped; ****p < 0.0001).

Ca^2+^ transients may be measured simultaneously with contractility. In Figure 6, we present data from measurements of changes in Ca^2+^ activity within mouse and rat cardiomyocytes loaded with Fura-4F. Example Ca^2+^ transients for rat and mouse are presented in Figures 6A,B, respectively. The peak heights of the Ca^2+^ transients (delta F/F0) are moderately clustered in mouse and rat cells (ICC, 26.6%; Supplementary Figures 5A,D). Population and hierarchical estimated means indicate little difference between the F/F0 values for rat and mouse. Neither the population nor hierarchically estimated means are statistically different (population, *p* = 0.23; estimate, *p* = 0.8 df 14.9 + 13.5; Figure 6C). Intriguingly, the TTP90 is once again the least clustered parameter (ICC, 2.7%), but it is still suggested to require a hierarchical approach (Supplementary Figures 5B,E). The TTP90 is longer in mouse than in rat; this is statistically significant according to common (*p* < 0.0001) and hierarchical methods (*p* < 0.0001, df 14 + 10; Figure 6D). The data for the TTB90 parameter are moderately clustered (ICC, 13.4%; Supplementary Figures 5C,F). Like TTP90, TTB90 is longer in mouse than in rat. This is suggested to be significantly different by the common (*p* < 0.0001) and hierarchical methodologies (*p* < 0.0001, df 14 + 12; Figure 5E). In the guinea pig, the height of the Ca^2+^ transient (delta F/F0) is similar to mouse and rat, but the onset of Ca^2+^ release is slower, and the decline of Ca^2+^ is slower (Supplementary Figures 4H–J) in common with the contractile transients (Table 3).

**TABLE 3:** Baseline measurements of calcium in myocytes from mouse, rat, and guinea pig using Fura-4f.

			**Simple**			**Hierarchical**							

	**Prep.**	***n***	**F/F0**	**TTP90**	**TTB90**	**F/F0**	**TTP90**	**TTB90**	**Suit.**	***p* values**			
Rat	6	505	1.136 ± 0.003	0.017 ± 0.0002	0.193 ± 0.004	1.134 ± 0.013	0.017 ± 0.0004	0.198 ± 0.019	YYY	Rat vs. Mouse			
											F/F0	TTP90	TTB90
										Simple	0.157	<0.0001	<0.0001
										Hierarchical	0.802	<0.0001	<0.0001
Mouse	7	311	1.143 ± 0.004	0.021 ± 0.0004	0.411 ± 0.009	1.131 ± 0.014	0.022 ± 0.0005	0.418 ± 0.017	–	Mouse vs. Guinea Pig			
											F/F0	TTP90	TTB90
										Simple	<0.0001	<0.0001	<0.0001
										Hierarchical	–	–	–
Guinea Pig	1	75	1.196 ± 0.001	0.083 ± 0.006	0.564 ± 0.016	–	–	–	–	Guinea Pig vs. Rat			
											F/F0	TTP90	TTB90
										Simple	<0.0001	<0.0001	<0.0001
										Hierarchical	–	–	–

*Comparison of the mean and standard error of parameters of calcium handling measured by Fura-4f dye labeling of rat, mouse, and guinea pig cardiomyocytes. Advanced statistical tests are applied to mouse and rat populations to indicate statistically significant differences between the kinetic parameters. Results from a number of preparations are presented using simple statistics (mean ± standard deviation) and using hierarchical statistics [mean ± standard deviation and (ICC)]. The *p* values column presents the calculations of *p* using Student’s *t* test or the hierarchical method.*

### Morphological Analysis

A morphological analysis of the entire cell can be performed using the thumbnails acquired in the course of the analysis runs, allowing the derivation of several morphological parameters of interest. We present the values acquired for a population of control rat cells (Table 4). In Supplementary Figure 1, we present a comparison of rat and guinea pig cardiomyocyte morphologies. Between 50 and 100 cells from a single rat and a single guinea pig isolation were assessed. Although visually similar (Supplementary Figures 1A,B), the two cell types diverge in several respects. The sarcomere length of guinea pig cells is shorter than the rat (Supplementary Figure 1C). Guinea pig cells have a greater area (Supplementary Figure 1D) and perimeter length (Supplementary Figure 1E) than rat cells. They are also longer (Supplementary Figure 1F) and wider (Supplementary Figure 1G). Interestingly, it appears that rat cells are more linear (higher eccentricity) and less lobular (higher solidity) than guinea pig cells (Supplementary Figures 1H,I and Table 4). The higher linearity of rat cells may suggest that there is some evolutionary benefit to a higher aspect ratio for this species. From the perspective of solidity, these data suggest that rat cells form fewer branching contacts with their neighboring cells or disaggregate more fully upon isolation.

**TABLE 4:** Comparison of morphology of rat and guinea pig cells.

		**Mean**	**SEM**	***n***	***p* value**
Sarcomere	Rat	1.826	0.005	101	0.0001
	Guinea Pig	1.776	0.01	50	
Area	Rat	3687	85.96	101	0.0001
	Guinea Pig	4991	168.2	50	
Length	Rat	133.3	1.855	101	0.0001
	Guinea Pig	161.0	3.432	50	
Width	Rat	37.39	0.64	101	0.0002
	Guinea Pig	42.69	1.22	50	
Perimeter	Rat	386.9	6.91	101	0.0001
	Guinea Pig	498.9	10.64	50	
Eccentricity	Rat	0.956	0.002	101	0.5
	Guinea Pig	0.959	0.004	50	
Solidity	Rat	0.859	0.005	101	0.0001
	Guinea Pig	0.815	0.007	50	

*Comparison of basic morphological parameters of cardiomyocytes isolated from Rat and Guinea Pig acquired in the process of generating functional measurements on the CytoCypher and analyzed by our automated script and the CytoSpectre program. Analysis was performed with conventional statistical tests as only single preparations were used.*

### Assessment of Small Molecules That Act on Myocyte Contractility

**FIGURE 7:**
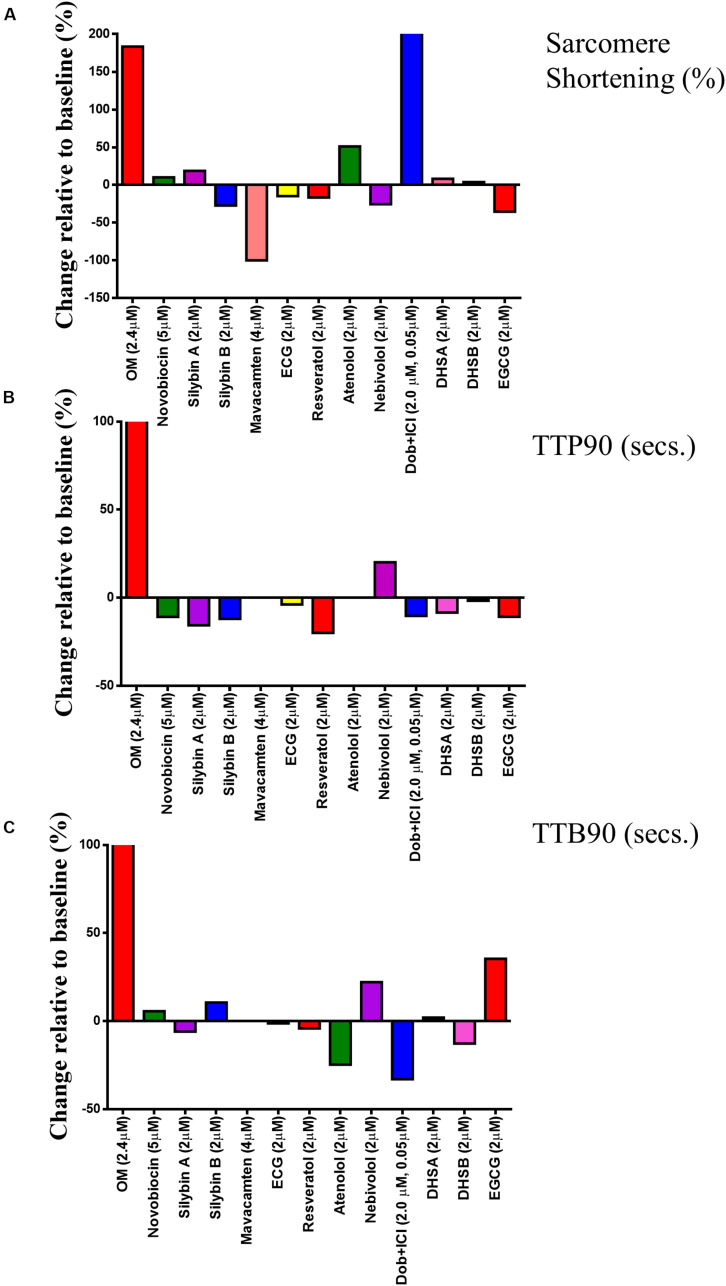
Single concentration screen for compounds with potential to affect myocyte contractility. The effects of 13 compounds were tested using a single preparation of rat myocytes. **(A–C)** show the fractional change in each parameter due to the compound: *y* = 100 × (mean with compound/mean baseline)-100. For original data, see Supplementary Figure 6. These data are constructed from a dataset of between 25 and 30 cardiomyocytes.

Using the CytoCypher HTS, it is now possible to dramatically speed up the measurement of the effects of agonists and antagonists on contractility and Ca^2+^ transients. Many measurements may be made on a single preparation of myocytes and so the confounding effects of inter-sample variability are reduced by performing a larger number of tests on the same preps, giving individual runs internal consistency. If technical replicates are performed, the aggregate data can be analyzed with hierarchical statistics; this improves the statistical power of the experiments by reducing the intrinsic variability of the samples, introduced mainly by preparation effects. As shown above, this dramatically improves the statistical power of measurements. Figure 7 shows an example of a simple drug screening protocol using a single rat myocyte preparation with several established β-agonists and antagonists, recently developed drugs that act on myocyte contractility and novel compounds that are proposed to have “recoupling” activity (Tadano et al., 2010; Papadaki et al., 2015; Sheehan et al., 2018). The 13 compounds were tested in about 5 h. Myocytes are kept at 4°C and plated onto laminin-coated dishes in three batches at approximately 2-h intervals to avoid run down in older preparations of cardiomyocytes. For each dish, a baseline is measured and then the solution is changed to one containing the drug. Most of the time is taken up with equilibration periods: if the compounds could be loaded in parallel in multi-well dishes, recording could be speeded up a further 5- to 10-fold (Juni et al., 2019).

In our analysis, myocytes with zero% shortening are included in the average to avoid bias (Figure 7A); however, they are excluded from the TTP90 and TTB90 averages since rate processes are only relevant when contraction occurs (Figures 7B,C). The results for individual cells are also presented in Supplementary Figure 6 for each of the three parameters. The result for dobutamine is as expected. Inotropic and lusitropic changes are evident with an increase in shortening amplitude accompanied by faster contraction (TTP90 less) and relaxation (TTB90 less). The myosin activator OM in contrast produces an increased mean contraction amplitude accompanied by slowing of both contraction and relaxation, as found previously, indicating that it enhances contractility by prolonging the contractile event (Woody et al., 2018; Juni et al., 2019). The myosin inhibitor Mavacamten reduces contractile amplitude to the point where so few myocytes are contracting that TTP90 and TTB90 cannot be measured as previously shown (Green et al., 2016). The effects of the other compounds were not particularly distinctive. Atenolol increased the% shortening and decreased TTB90 similar to dobutamine; this effect was observed in two further screens (see Supplementary Figure 7). Nebivolol and EGCG both increased TTB90 by about 20% but only the Nebivolol effect was consistently found. Nebivolol, ECG, and EGCG have been proposed as desensitizers, but may also have additional actions (Nebivolol as a beta blocker and EGCG as an inotrope; Feng et al., 2012; Stücker et al., 2017). None of these compounds showed a reduction of% shortening or reduction of TTB90 indicative of enhanced relaxation in our study. Most of the compounds proposed to affect the dobutamine response of mutant myocytes (“recouplers”: Silybin A and B, dehydrosilybin A and B, resveratrol, and novobiocin) had little effect on the baseline response in wild type as predicted (Papadaki et al., 2015; Wilkinson et al., 2015; Sheehan et al., 2018).

To test for reproducibility of the drug screening experiment, we repeated it three times and analyzed the data with hierarchical statistics as well as simple statistics (Supplementary Figure 7A). In comparison to the conventional approach, the SEM adjusted with hierarchical statistics were much larger, reducing the chances of false-positive results. Dobutamine, OM, and Mavacamten effects remained significant, and significant changes were uncovered in SilybinB, SilybinA, ECG, Atenolol, DHSA, and EGCG. Moreover, drugs such as Resveratrol and EGCG demonstrated opposing relationships in the change of contractile force relative to baseline conditions before and after adjusting with hierarchical statistics. Supplementary Figure 7B illustrates the ratio change between treatment and baseline contractility values and were subsequently expressed as a percentage.

We conclude that this type of screen can identify effects of small molecules upon the relevant parameters of myocyte contractility rapidly and does not produce false positives, as is evident when repeats of the screen are compared (Supplementary Figures 7A,B). An initial screen could be performed this way, followed up by more extensive tests of the compounds that can be followed up by more extensive tests of the compounds that show promise; moreover, the screen uses only a small amount of solution (2.5 ml), but a much smaller volume is possible, enabling rare or expensive compounds to be assessed.

**FIGURE 8:**
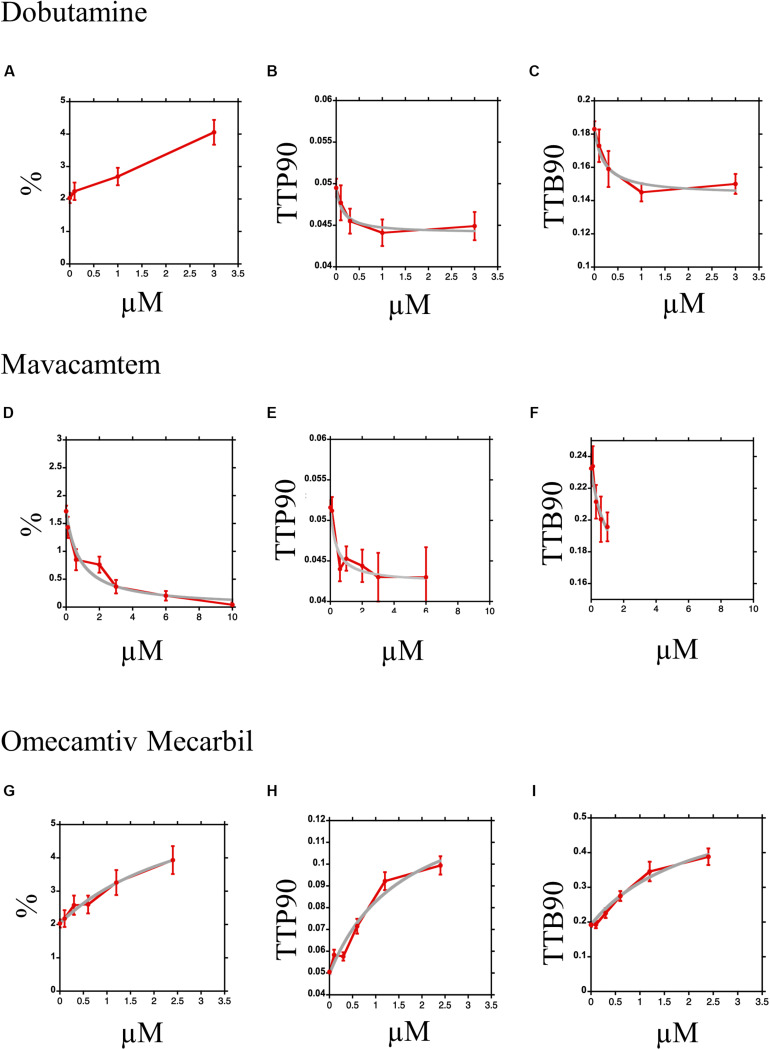
Dose–response curves for Dobutamine, Omecamtiv Mecarbil, and Mavacamten. **(A–C)** Dose–response curve for myocyte contraction in the presence of Dobutamine and 0.05 μM ICI 118,551. The mean and standard error are plotted in red; the data for TTP90 and TTB90 are fitted to the equation *y* = *y*0–[Bm.(dobu)/(EC50 + (dobu))] in gray. The % shortening parameter could not be fitted. **(D,E)** Dose–response curve for myocyte contraction in the presence of Omecamtiv Mecarbil. The mean and standard error are plotted in red; the data for all three parameters are fitted to the equation *y* = *y*0 + [Bm.(OM)/(EC50 + (OM))] in gray. **(G–I)** Dose–response curves for myocyte contraction in the presence of Mavacamten. The mean and standard error are plotted in red; the data for all three parameters are fitted to the equation *y* = *y*0–[*y*0.(mava)/(EC50 + (mava))] in gray.

Dose–response curves are readily constructed using the CC-HTS. With a single preparation of cells, we can obtain two dose–response curves in 5 h, tracking 10–20 cells per condition. We demonstrate here dose–response curves for three contrasting drugs that act upon myocyte contractility. Figures 8A–C show the dose–response curve for dobutamine in the presence of 50 nM ICI 118,551. The increase in% shortening was almost linear and did not satisfactorily fit a simple binding equation (Figure 8A); however, TTP90 (Figure 8B); and TTB90 (Figure 8C) showed simple saturation kinetics with EC50 of 0.14 ± 0.06 μM and 0.21 ± 0.09 μM, respectively. Scatter plots showing values of individual myocytes can be found in Supplementary Figures 8A–C.

Figures 8D–F show the inhibition of contractility by Mavacamten. Percent shortening was well fitted to a simple binding equation with EC50 of 0.8 ± 0.16 μM (Figure 8D). TTP90 was reduced by 18% with an EC50 of 0.37 ± 0.20 μM; the significance of the fit is reduced since the number of contracting myocytes diminished rapidly at the higher concentrations (Figure 8E). For TTB90, an EC50 of 0.9 ± 0.9 μM was estimated from the small number of data points available (Figure 8F). Scatter plots showing values of individual samples can be found in Supplementary Figure 8.

All three parameters were increased by OM (Figures 8G–I); however, at concentrations above 3 μM, we found that the majority of cells became hypercontractile and rapidly rounded up. Although the concentration range was lower than ideal, the data fits to a simple binding equation were obtained. The curve fitting indicated that OM increased% shortening by 216% (Figure 8G), TTP90 by 175% (Figure 8H), and TTB90 by 202% (Figure 8I) with EC50 of 3.1 ± 1.3 μM, 1.8 ± 0.9 μM, and 2.2 ± 0.9 μM respectively. Scatter plots showing values of individual samples can be found in Supplementary Figure 8.

## Discussion

The introduction of a high-throughput method for measuring myocyte contractility and Ca^2+^ transients has the potential to revolutionize the study of cardiomyocyte pharmacology, physiology, and pathophysiology. We have shown that it is now possible to collect rapidly large amounts of data from a single myocyte preparation enabling comparisons to be made using simple statistics. This is ideal for drug screens and dose–response studies. The quantity of data collected allows robust hierarchical statistical analysis that is of value when comparing different cell preparations. The speed of data collection means unstable myocyte preparations can now be more fully exploited.

Other researchers, for example, Harmer et al. (2012), have attempted to increase the productivity of analyses of adult cardiomyocytes, but the CytoCypher represents a comprehensive system for this. Myocardial preparations of intermediate complexity have existed for longer than the isolated cardiomyocyte approach; each has advantages and disadvantages. Papillary muscles and ventricular strips have been employed in organ bath experiments for decades and have allowed researchers to assess the effect of pathological and pharmacological interventions on mechanically loaded preparations (Frisk et al., 2016). However, these preparations are not illustrative of specific layers of the compacted working, ventricular myocardium. More recently, researchers have tried to produce engineered heart tissue constructs from induced pluripotent stem cells (Hirt et al., 2014). Despite being contractile and capable of producing physiologically relevant force, the phenotype of the cells is not consistent with preparations from native hearts. The preparation of ultra-thin myocardial slices using microtomy offers a useful approach to assess single cells and their conduction properties from bona fide ventricular myocardial tissue (Watson et al., 2019a, b). Slice preparations preserve the multi-cellularity, architecture, and physiological environment of the ventricular tissue. These preparations can be manipulated mechanically and survive well in culture. As these preparations are only a few cells thick, the observer is still able to interrogate the structural phenotype of individual cells within a layer using conventional confocal microscopy techniques. Currently, this methodology is limited to larger rodents, obviating the analysis of tissue from mouse models and so precluding the acquisition of important data from hearts manipulated with genetic approaches and tools.

Until now, myocyte contraction measurements have been low-throughput assays. They require multiple heart isolations to collect sufficient data that generate well-powered experiments to distinguish changes due to treatments from inter-prep variability. The CC-HTS system avoids most of these problems by measuring contractility (and Ca^2+^) in large numbers of myocytes from a single animal in a short time while cells are viable. Richer datasets are produced that are more representative of the entire population of cells present in the native myocardial sample.

This has enabled us to address the question of how to handle the statistics of inter-preparation and within-preparation variability. By analysis of many hundreds of myocytes, the problem of psuedoreplication leading to false-positive results when data from different preparations are combined is laid bare. This clearly has implications for many current studies that do not take this into account. HTS allows us to collect enough data to confirm or deny differences between treatments or between cell types with the appropriate statistical routine to ensure the statistical power is adequate.

High-throughput systems reduce the pseudoreplication problem since all measurements can be made on one preparation within a short period of time, thus legitimizing the use of simple statistics (Juni et al., 2019). Examples of such measurements are shown for single concentration drug screens and dose–response curves. The HTS method also allows us to study rare or unstable myocytes, such as myocytes derived from human heart material that may have been impossible to measure previously. From a practical point of view, with increased measurement speed, it now becomes possible (in some species/conditions) to study almost all the myocytes in a single preparation, which is a major advantage when myocytes come from rare or expensive sources, such as transgenic mice.

We propose two experimental areas in which the deployment of high-throughput analyses of isolated cardiomyocytes will be advantageous. Firstly, in drug screening where the inter-sample variation is extremely problematic, we propose that the increase in productivity should be used to screen multiple drugs. Secondly, where the baseline characteristics of populations of animals, such as those used for disease modeling or genetic mutants where inter-preparation effects are unavoidable, the high-throughput approach can be used in tandem with advanced statistical approaches to eliminate false-positive effects.

## Conclusion

The introduction of HTS methodology for myocytes has increased the speed of measurement dramatically and improved statistical power while reducing the number of myocyte preparations needed for a study.

## Data Availability Statement

The datasets generated for this study are available on request to the corresponding author.

## Ethics Statement

The studies involving human participants were reviewed and approved by United Kingdom Human Tissue Authority and Research Ethics Committees. The patients/participants provided their written informed consent to participate in this study. The animal study was reviewed and approved by Imperial College AWERB – Imperial College London, United Kingdom.

## Author Contributions

PW and SM conceived the study, performed experiments, and authored the manuscript. ST and AF performed experiments. KM assisted in the preparation of the manuscript. All authors contributed to the article and approved the submitted version.

## Conflict of Interest

The authors declare that the research was conducted in the absence of any commercial or financial relationships that could be construed as a potential conflict of interest.
