# Supplementary material for: Approaches to High-Throughput Analysis of Cardiomyocyte Contractility
Source: Front Physiol. 2020 Jul 8;11:612. doi: 10.3389/fphys.2020.00612 (PMC7362994; doi:10.3389/fphys.2020.00612)
Supplement: Supplementary file 2 [file Data_Sheet_1.PDF]

Supplementary Figure 1

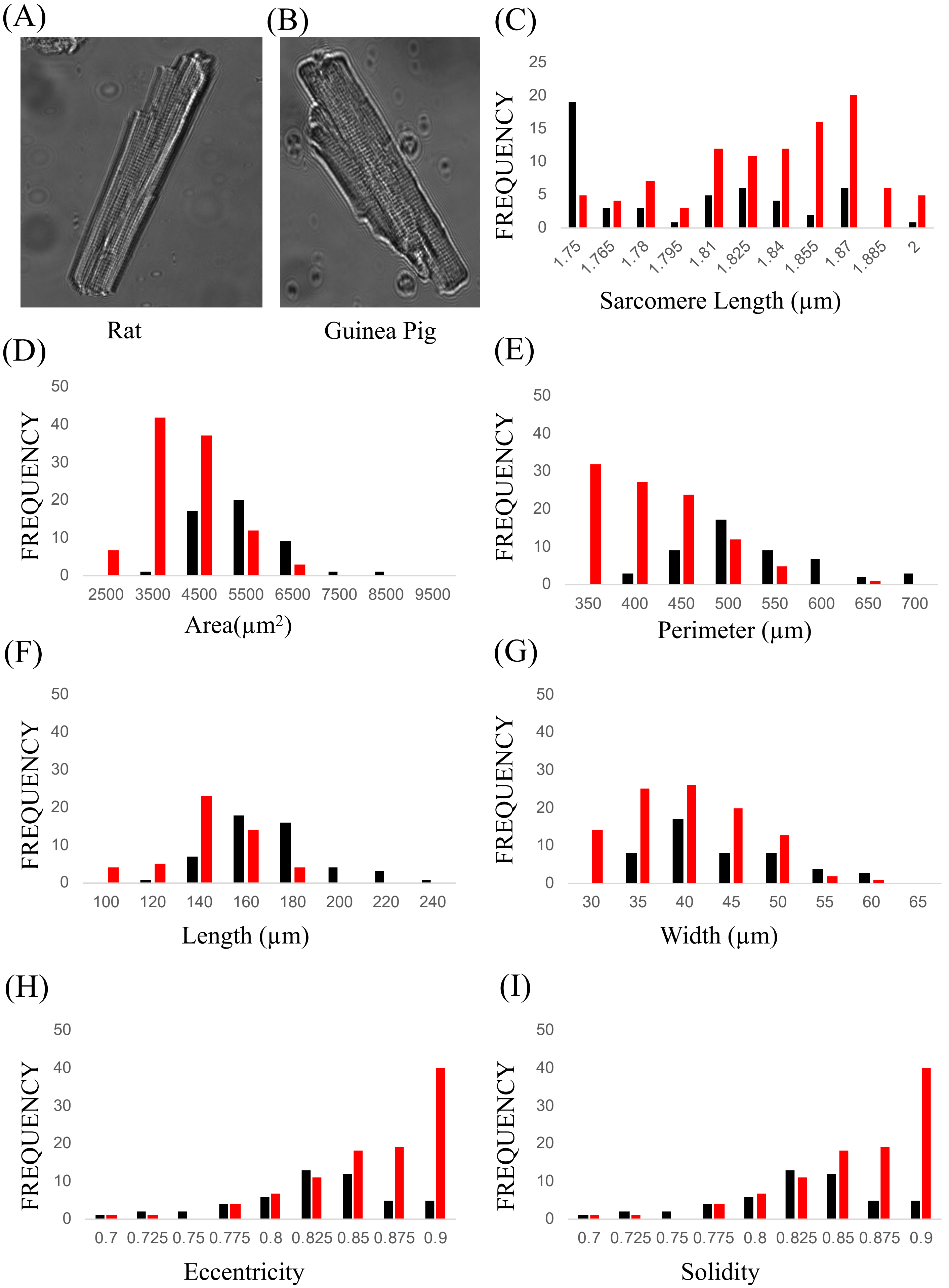

Supplementary Figure 1

A) Example images of a rat and B) a guinea pig cardiomyocyte. Comparisons of the frequency distributions of C) sarcomere length, D) area, E) perimeter, F) length, G) width, H) eccentricity and F) solidity for 100 rat (red) and 50 guinea pig cells (black).

Supplementary Figure 2

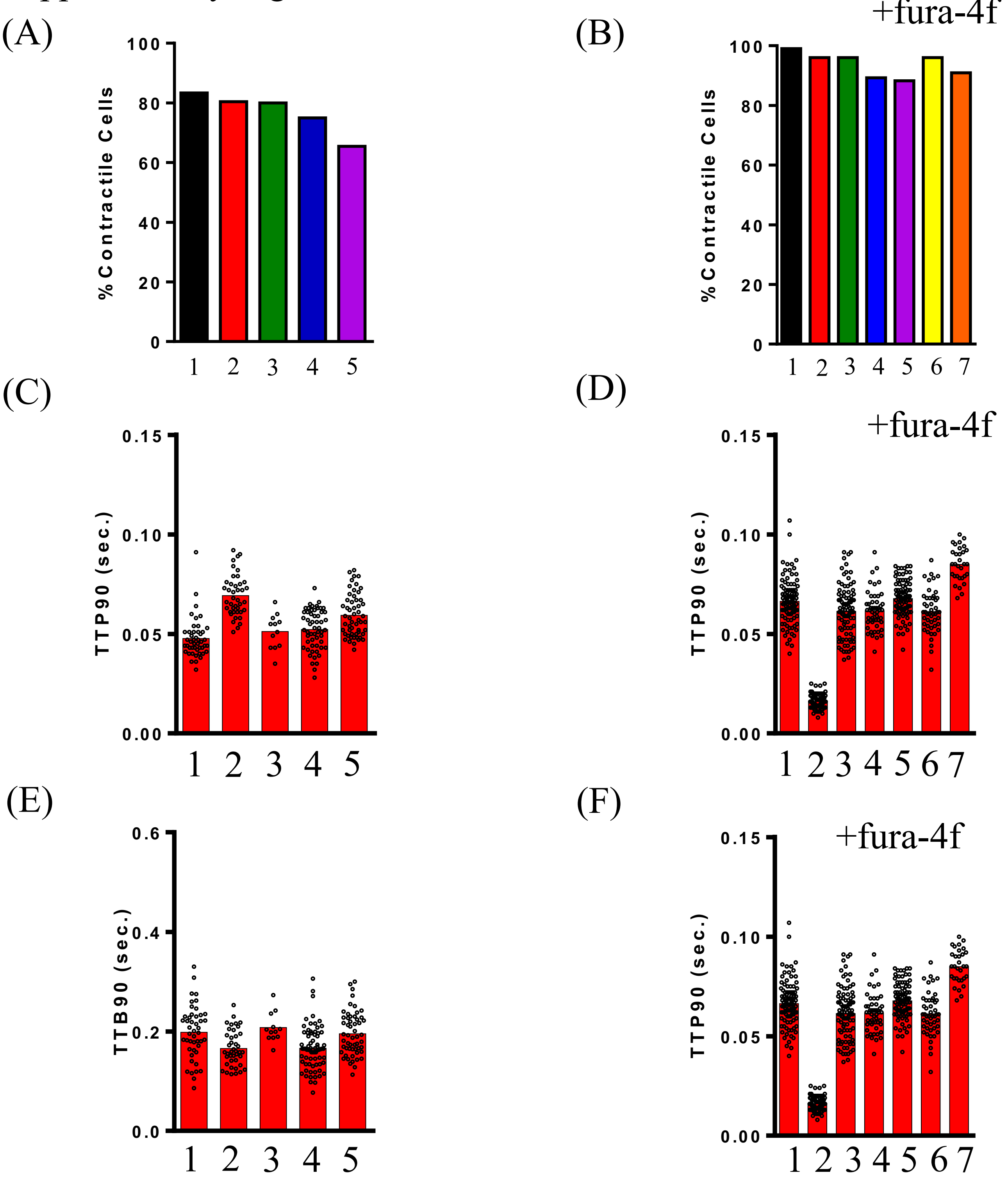

Supplementary Figure 2

A) Comparison of the percentage of contractile cells (as defined by CytoCypher automated collection criteria) in freshly isolated rat preparations.

B) Comparison of the percentage of contractile cells following the loading of cells with the Fura-4f Ca^{2+}-indicator dye in separate preparations from different rats.

C, D) Histogram displaying measurements of the time to peak sarcomere shortening (TTP90) in multiple preparations of rat cardiomyocytes without and with fura-4f loading.

E, F) Histograms displaying measurements of the time to baseline sarcomere shortening (TTB90) in multiple preparations of rat cardiomyocytes without and with fura-4f loading.

Supplementary Figure 3

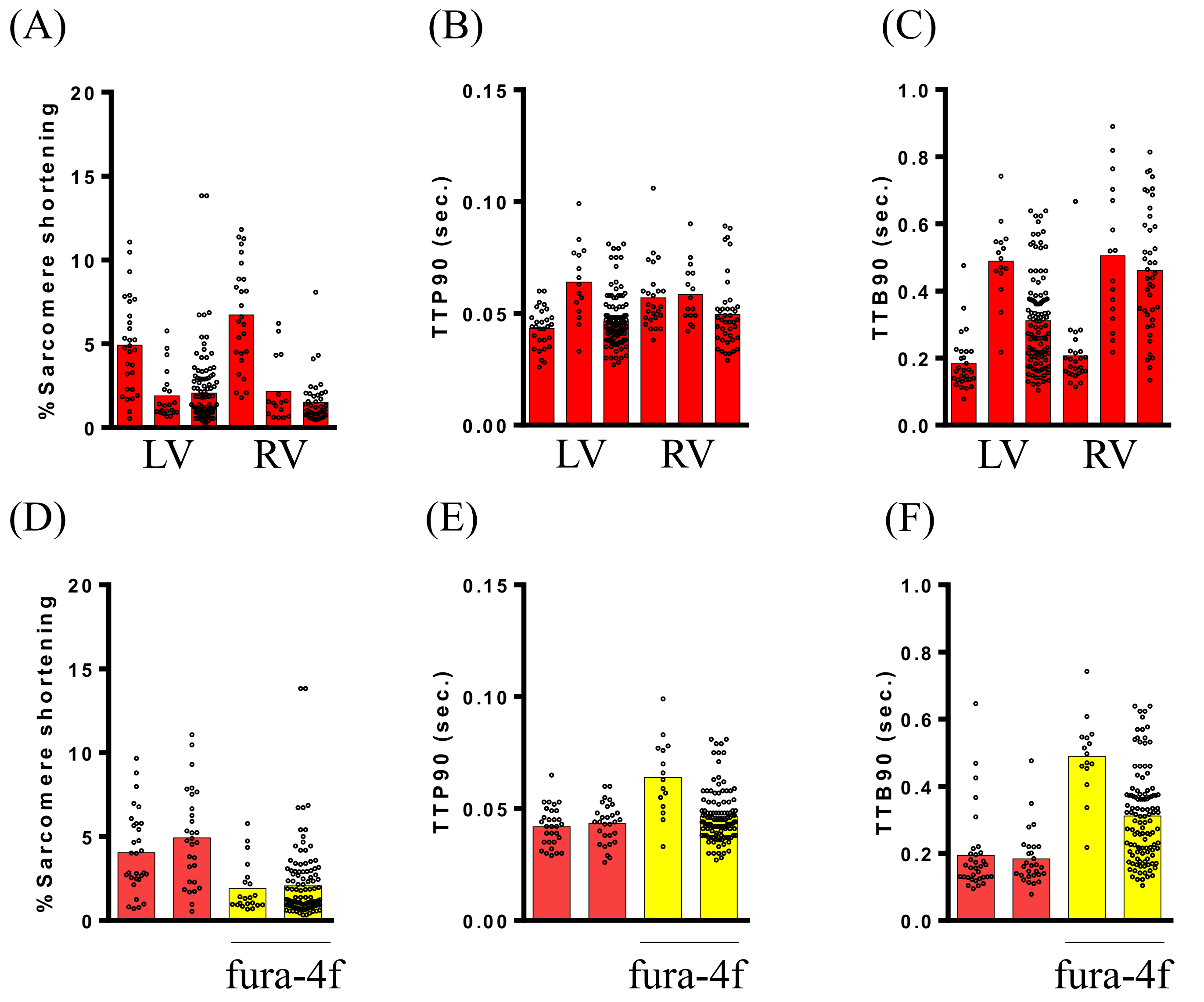

<u>Supplementary Figure 3</u>

A) Histogram presenting the baseline sarcomere shortening of cardiomyocytes derived from the right (RV) and left ventricles (LV) of mice, in three separate preparations.

B) The TTP90 of the sarcomere shortening of cardiomyocytes from left and right ventricle

C) The TTB90 from the same preparations

D) Histogram representing the mean sarcomere shortening of mouse cells isolated from the whole heart without (pink bars) and with fura-4f loading (yellow bars)

E) Histogram representing the mean and standard error of TTP90 of sarcomere shortening from the data set in D) Control cells (pink bars) and cells loaded with fura-4f (yellow bars)

F) Histogram representing the mean and standard error of TTB90 of sarcomere shortening from the data set in D) Control cells (pink bars) and cells loaded with fura-4f (yellow bars)

Supplementary Figure 4

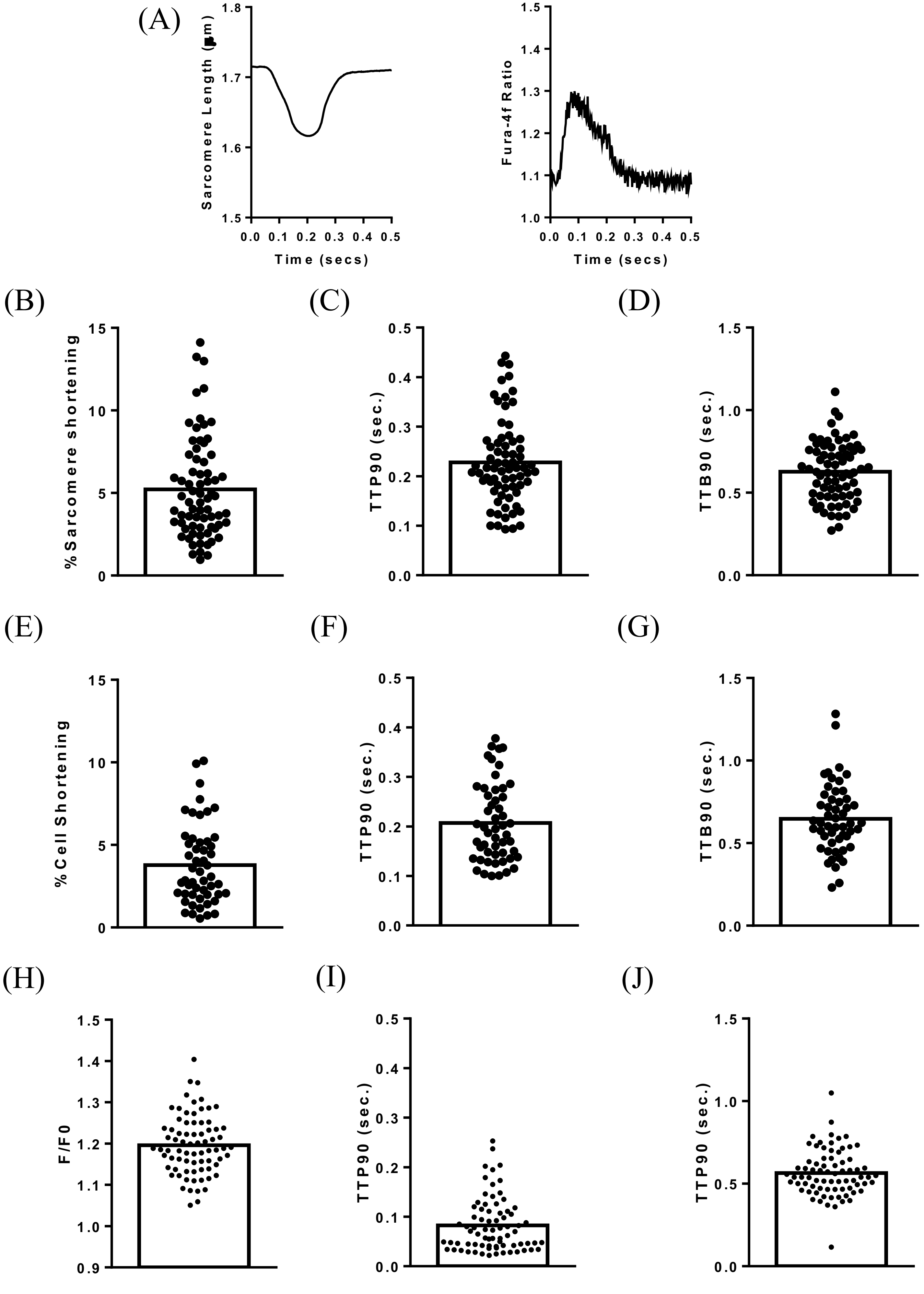

Supplementary Figure 5

A) Example traces of sarcomere shortening and fura-4f response from a guinea pig cardiomyocyte paced at 0.5Hz

B) Histogram and dot plot representing the baseline sarcomere shortening of guinea pig ventricular cardiomyocytes stimulated 0.5Hz

C) Histogram and dot plot representing the TTP90 of sarcomere shortening

D) Histogram and dot plot representing the TTB90 of sarcomere shortening of the same set of cells

E) Histogram and dot plot representing the percentage cell shortening of the guinea pig cardiomyocytes stimulated at 0.5Hz

F and G) Histogram and dot plots representing the TTP90 and TTB90 of cell shortening in the same data set.

H) Histogram and dot plot representing the F/F0 of calcium transient of the guinea pig cardiomyocytes stimulated at 0.5Hz

F and G) Histogram and dot plots representing the TTP90 and TTB90 of calcium transient in the same data set.

Supplementary Figure 5

RAT

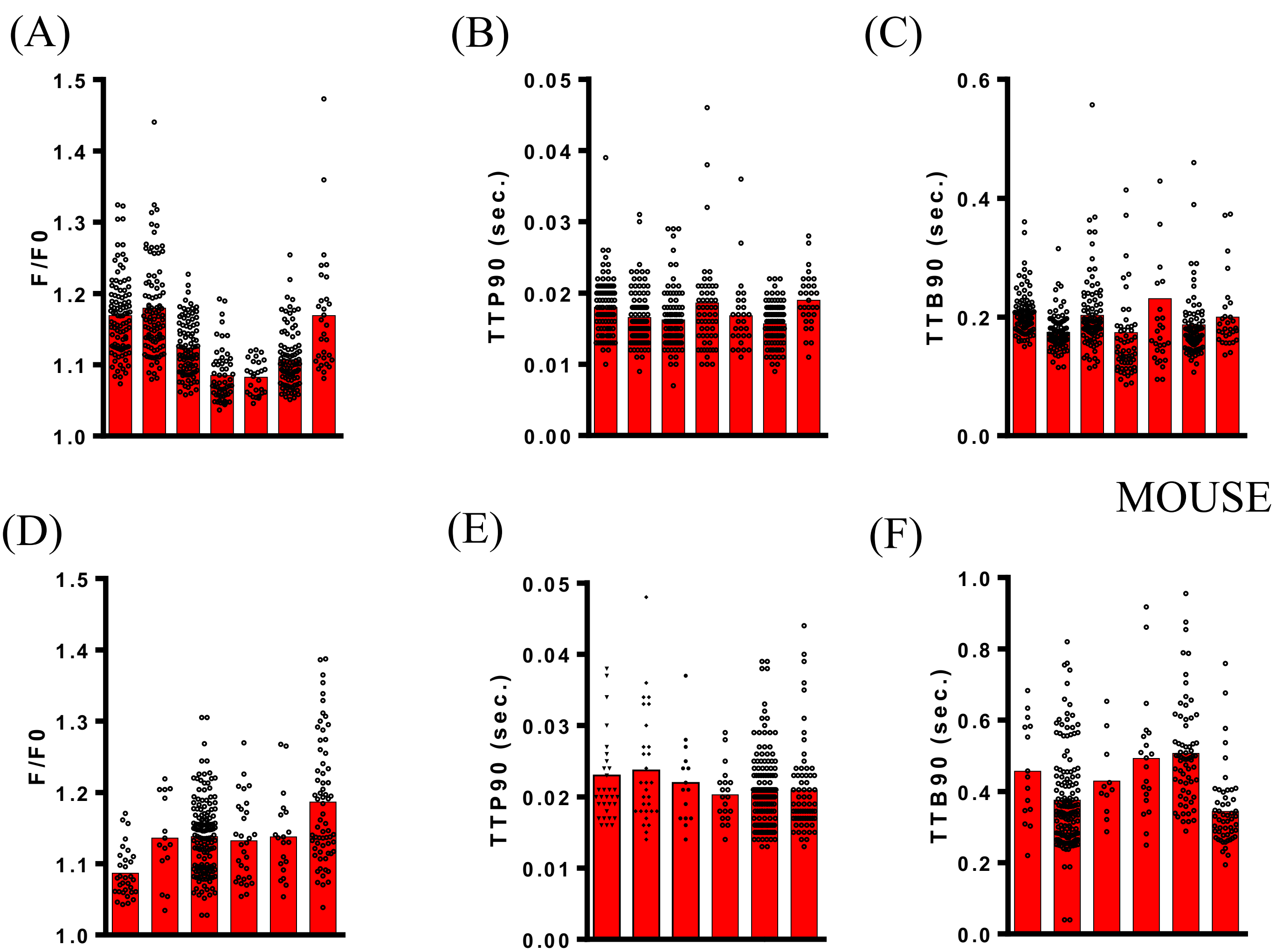

<u>Supplementary Figure 5</u>

A) Histogram representing the mean amplitude (F/F0) of calcium transient in rat cardiomyocytes derived from seven separate preparations

B) Histogram representing the mean TTP90 of calcium transients in rat cardiomyocytes derived from seven separate preparations

C) Histogram representing the mean TTB90 of calcium transients in rat cardiomyocytes derived from seven separate preparations

D) Histogram representing the mean amplitude (F/F0) of calcium transient in mouse cardiomyocytes derived from six separate preparations

E) Histogram representing the mean TTP90 of calcium transients in mouse cardiomyocytes derived from six separate preparations

F) Histogram representing the mean TTB90 of calcium transients in mouse cardiomyocytes derived from six separate preparations

Supplementary Figure 6

(A)

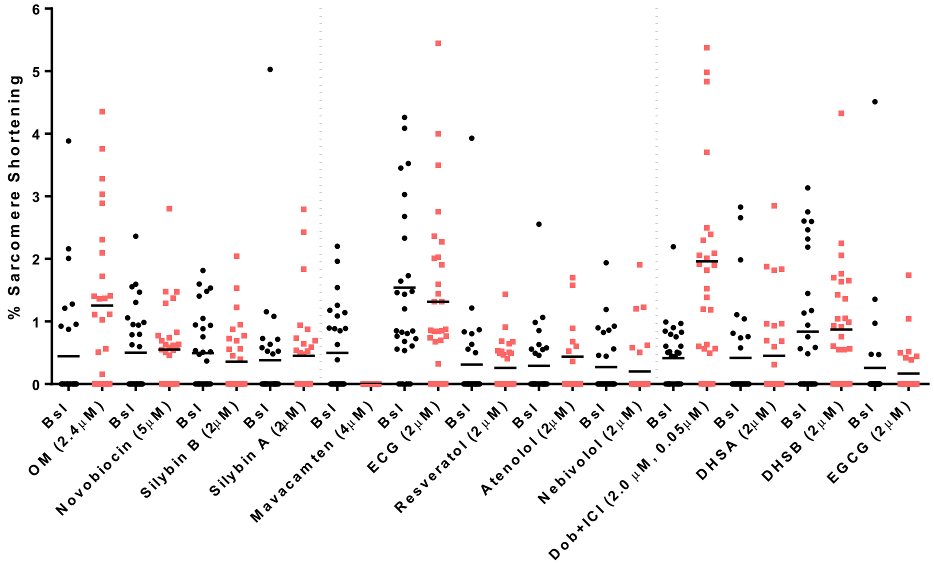

(B)

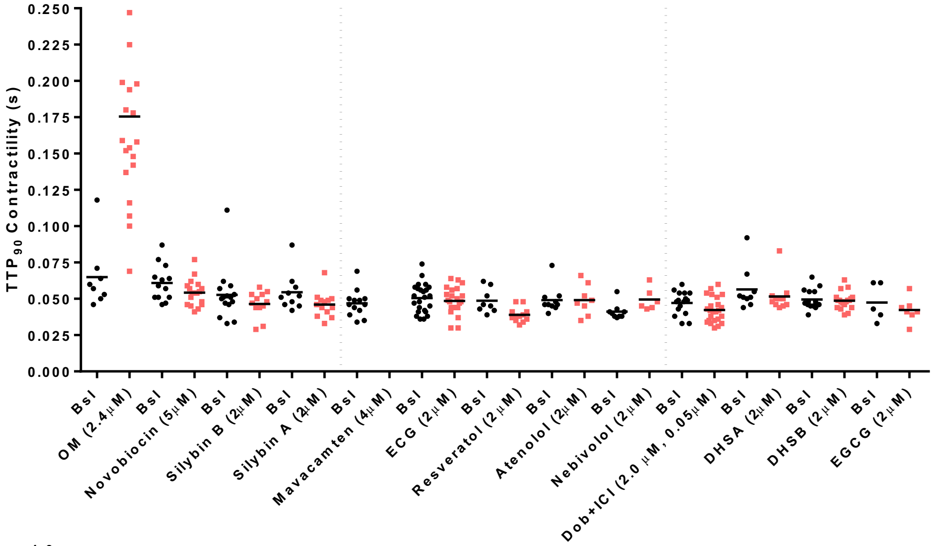

(C)

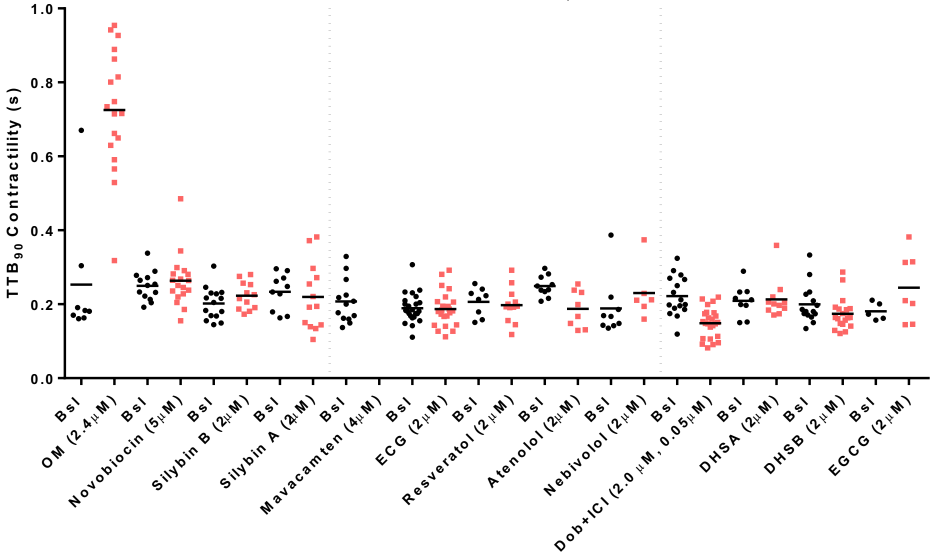

Supplementary Figure 6

Dot plots presenting the results of our 13 compound screen, 25-30 myocytes were assayed at baseline and then again in the presence of the compound used to construct Figure 7. a) The % shortening, b) TTP90 and c) TTB90 were calculated for each myocyte. Each point in the plot represents an individual myocyte with the sample mean shown.

Supplementary Figure 7A

Drug	n=cell#	Corrected n d	Corrected d.f	T test (P value)			Intraclass correlation (ICC) (%)			Hierarchical Clustering (P value)		
	Format layout<br><br>%SS. (Baseline:Treated)<br>TTP90 (Baseline:Treated)<br>TTB90 (Baseline:Treated)			%SS	TTP_{90}	TTB_{90}	%SS	TTP_{90}	TTB_{90}	%SS	TTP_{90}	TTB_{90}
Omecamtiv Mercarbil	90:69<br>30:33<br>30:33	27:27<br>7:7<br>6:6	5.6;6.7<br>6.7;6.3<br>6.7;6.4	0.1062	< 0.0001	< 0.0001	9.8	37.9	47.9	0.3572	0.0874	0.0858
Novobiocin	71;79<br>44;51<br>44;51	43:45<br>25:26<br>22:23	5.4;4.4<br>6.9;6.0<br>6.9;5.7	0.4152	0.0008	0.2718	5.5	9.1	10.7	0.5433	0.0532	0.4791
Silybin B	71;81<br>36;43<br>36;43	23:23<br>-<br>16:16	5.4;4.9<br>-<br>6.2;5.5	0.9705	0.0402	0.7597	11.6	0	16	0.8495	-	0.9427
Silybin A	72;85<br>31;45<br>31.45	4:4<br>-<br>11:11	5.9;5.8<br>-<br>6.8;6.0	0.5249	0.0003	0.1755	64.	2.4	24.7	0.5498	-	0.6332
Mavacamten	72;80<br>36;13<br>36;13	14:14<br>14:12<br>-	6.1;5.2<br>4.2; 6.4<br>-	< 0.0001	0.7484	< 0.0001	2	17.9	0	0.0265	0.7827	-
ECG	75;81<br>50;57<br>50;57	-<br>-<br>33:33	-<br>-<br>5.7;5.1	0.0138	0.0073	0.3698	0.4	2.7	7.2	-	-	0.014
Resveratrol	70;86<br>22;45<br>22;57	5:5<br>10:11<br>16:19	5.9;5.7<br>5.8;4.2<br>7.8;4.7	0.2844	0.0657	0.0652	53.8	24.2	13.9	0.8333	0.3613	0.2596
Atenolol	79;79<br>19;32<br>19;32	10:10<br>-<br>23:27	5.8;5.8<br>-<br>6.7;4.0	0.0453	0.0283	0.0001	28.3	1	8.1	0.4016	0.0716	0.0285*
Nebivolol	68;80<br>20;38<br>20;38	6:6<br>-<br>11:12	6.0;5.7<br>-<br>8.5:6.0	0.0147	0.0927	0.3986	44.5	2	22.2	0.652	0.2043	0.8915
Dob ICI	73;81<br>34;63<br>34;63	69:71<br>-<br>33:38	4.8;4.4<br>-<br>8.9;5.0	< 0.0001	0.0132	< 0.0001	3	0	6.3	0.0245	-	0.0109*
DHSA	73;81<br>27;46<br>27;46	8:8<br>-<br>12:13	5.9;5.7<br>-<br>7.2;5.6	0.1751	0.036	0.2299	33.8	6.1	21.3	0.8338	-	0.5666
DHSB	72;81<br>40;47<br>40;47	10:10<br>-<br>14:14	6.1;5.6<br>-<br>6.2;5.7	0.071	0.3175	0.1363	29	0	18.4	0.3114	-	0.4886
EGCG	70;81<br>16;39<br>16;39	6:6<br>-<br>8:9	6.2;5.8<br>-<br>7.9;6.0	0.7215	0.0328	0.4767	45.4	0	29.2	0.7607	-	0.9737
Cilostamide	193;2<br>18<br>161;1<br>57<br>161;1<br>57	14;14<br>13;13<br>-	6.2;6.3<br>5.8;5.8<br>-	<0.0001	<0.0001	<0.0001	20.1	21.4	0	0.0689	0.1189	-
Rolipram	193;2<br>03<br>161;9<br>2<br>161;9<br>2	21;21<br>17;17<br>23;22	5.8;5.7<br>5.5;6.2<br>4.6;5.4	<0.0001	0.1633	0.1284	13.7	16.3	12.5	0.5069	0.635	0.938
Isoprenaline	193;1<br>42<br>161;1<br>14<br>161;1<br>14	13;13<br>5;5<br>7;7	5.6;5.8<br>5.8;5.9<br>5.7;5.8	<0.0001	<0.0001	<0.0001	21.7	52.7	40.4	0.2342	0.4762	0.2726

Supplementary Figure 7A The effect of applying t-test or hierarchical statistics to study drug screens from 3 biological replicates (n=3). Contractile parameters following individual drug treatments were compared to respective baseline values through t-tests or hierarchical statistics. Significant values indicated in red. Layout for the number of cells and d.f is represented in the format (%SS, TTP90, TTB90), with each row representing a single parameter. Within each row is (baseline # number : drug treated # number). The d.f were corrected (if necessary) with the code supplied by Sikkel et al., 2017 in RStudio. The d.f excluded from correction were determined by the code and are highlighted in yellow. If correction was deemed unnecessary due to low ICC, the hierarchical statistics can be ignored, as indicated by '-', and the P value can be obtained from the T test. Equation to correct for effective sample size is (n*m)/[1+(m-1*ICC)].

Supplementary Figure 7B

(A)

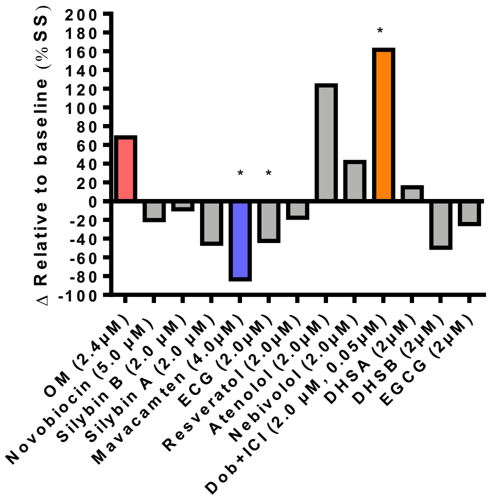

(B)

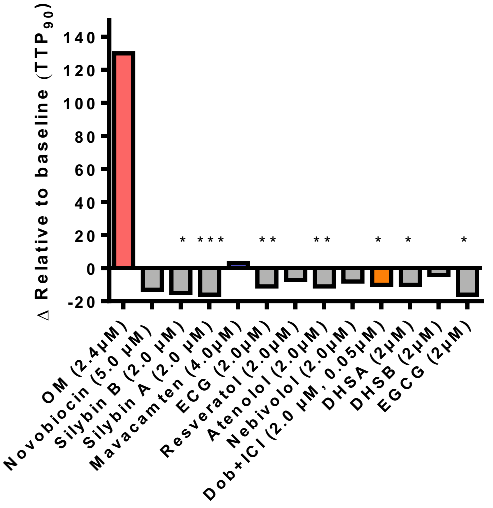

C)

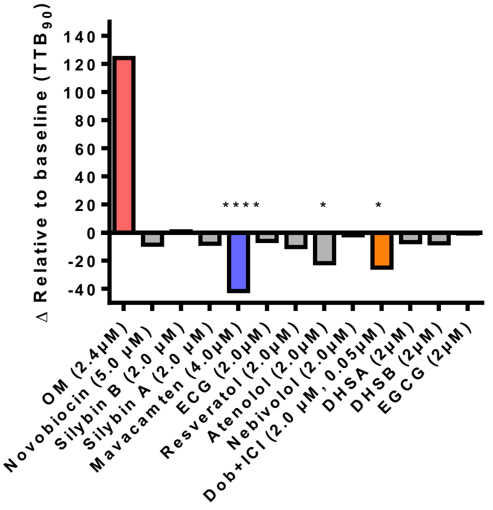

Figure 7B The effect of small molecule drugs on rat cardiomyocyte contractility. Data presents the relative changes in parameters for three biological replicates corrected with hierarchical statistics. The ratio changes in contractility parameters between drug treated and untreated cardiomyocytes are expressed as percentages. Contractile parameters include %SS, TTP90 and TTB90. Internal controls Omecamtiv Mercarbil, Mavacamten, Dobutamine indicated in red, purple & orange respectively. Hierarchical analysis, (n=3) *P<0.05 **P<0.01 ***P<0.001 ****P>0.0001

Supplementary Figure 8

(A) Dobutamine

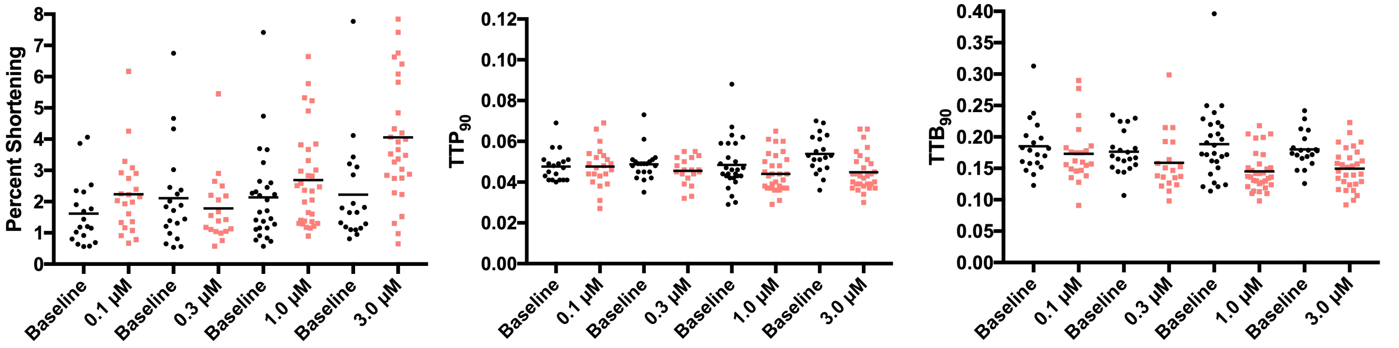

(B) Mavacamten

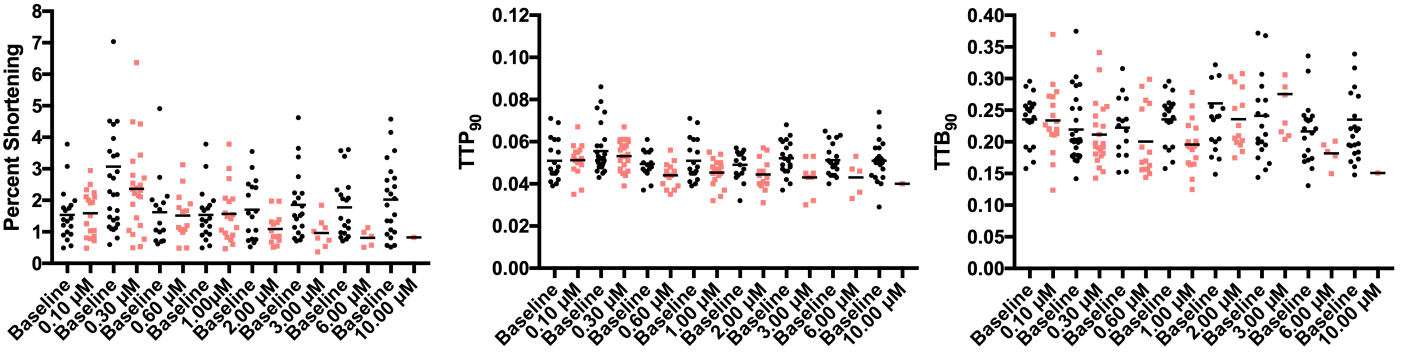

C) Omecamtiv

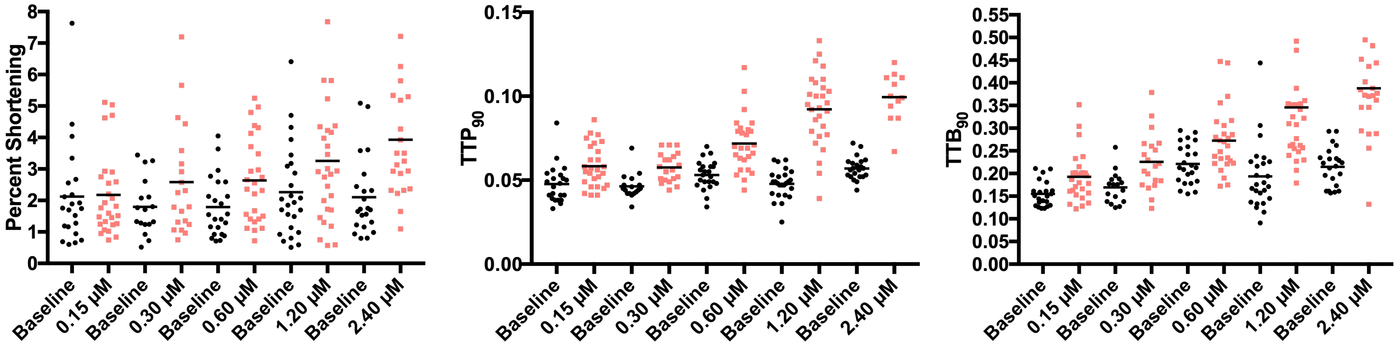

Supplementary Figure 8

A) Scatter plot showing a dose-response experiment following treatment with Dobutamine and 0.05µM ICI 118, 551. 20-30 myocytes are measured using automatic tracking. Plots show the experimental results for % shortening, TTP90 and TTB90. Black is baseline and red is measurements with dobutamine on the same dish of myocytes. Each Dobutamine concentration used a fresh dish of myocytes.

B) Scatter plot showing a dose-response experiment following treatment with Mavacamten. 20-30 myocytes are measured using automatic tracking. Plots show the experimental results for % shortening, TTP90 and TTB90. Black is baseline and red is measurements with Mavacamten on the same dish of myocytes. Each Mavacamten concentration used a fresh dish of myocytes.

C) Scatter plot showing a dose-response experiment following treatment with Omecamtiv Mecarbil. 20-30 myocytes are measured using automatic tracking. Plots show the experimental results for % shortening, TTP90 and TTB90. Black is baseline and red is measurements with Omecamtiv Mecarbil on the same dish of myocytes. Each Omecamtiv Mecarbil concentration used a fresh dish of myocytes.
